# Oscillatory and Aperiodic Contributions to EEG Event‐Related Time‐Frequency Metrics During Cognitive Control and Reinforcement Processing: A Registered Report

**DOI:** 10.1111/psyp.70073

**Published:** 2025-06-03

**Authors:** Eric Rawls, Scott R. Sponheim

**Affiliations:** ^1^ Department of Psychology University of North Carolina Wilmington Wilmington North Carolina USA; ^2^ Department of Psychiatry and Behavioral Sciences University of Minnesota Minneapolis Minnesota USA; ^3^ Minneapolis Veterans Affairs Health Care Services (VAHCS) Minneapolis Minnesota USA

**Keywords:** 1/f time frequency, alpha, aperiodic, cognitive control, mediofrontal theta, oscillations, reinforcement processing

## Abstract

Brain oscillations, or rhythms, coordinate communication across distributed brain networks. These rhythms provide a foundation for the brain network interactions required for cognition. Oscillations coexist with non‐rhythmic background aperiodic activity that forms a characteristic 1/f pattern in power spectra. Aperiodic brain activity is associated with cognition and can confound the detection of oscillations. In this Registered Report, we applied time‐resolved spectral parameterization to EEG recordings during two common cognitive tasks. Neural dynamics recorded during many cognitive paradigms show similar patterns, including synchronization of mediofrontal theta (4–8 Hz) and desynchronization of posterior alpha (9–13 Hz) and central beta (15–30 Hz). Our results indicate that common task time‐frequency signatures, including mediofrontal theta synchronization and parietal alpha desynchronization, can be attributed primarily to neural oscillatory phenomena. Intriguingly, we uncover evidence of stimulus‐locked aperiodic power changes, which are responsive to the need for cognitive control and to reinforcement processing. Furthermore, aperiodic power correlated strongly with non‐baseline‐corrected total power estimates, and whereas oscillatory power correlated strongly with portions of baseline‐corrected power estimates, it failed to correlate with other portions of baseline‐corrected power. Finally, after baseline correction, aperiodic correlations with TF power remain high. These results indicate two primary outcomes. First, task TF signatures in theta and alpha bands reflect primarily parameterized oscillations. Second, aperiodic activity is time‐dependent during cognitive processing, and these dynamics are not accounted for by baseline correction.

## Abstract 2

Event‐related EEG time‐frequency is assumed to reflect brain oscillations, but aperiodic activity can confound measuring neural oscillations. We show that spectral features in theta and alpha bands are essentially oscillatory while revealing aperiodic event‐related dynamics and condition differences during the need for control and reinforcement processing. Event‐related EEG features are not fully oscillatory, even after baseline correction, showing the need for enhanced analyses to study oscillations without aperiodic confounds.

## 1 Introduction

Oscillations coordinate communication across distributed brain networks, providing a foundation for the dynamic brain network interactions required for cognition and action (Voytek et al. 2015; Ward 2003). Brain oscillations appear as rhythmic waves that are often visible in neural recordings with the naked eye. The most prominent of these include the theta rhythm in the rodent hippocampus (Buzsaki 2006) and the alpha rhythm in human EEG (Nunez and Srinivasan 2006b). Brain oscillations coexist with background aperiodic activity which is not rhythmic and forms a characteristic 1/f pattern in power spectra, with lowest frequencies carrying the highest power. The entire neurophysiological source of this background activity is unknown, but it is potentially related to neurotransmitter balance in the brain (Gao et al. 2017). Aperiodic brain activity has important relationships with attention and cognitive control (Gyurkovics et al. 2022; Ostlund et al. 2021; Waschke et al. 2021; Zhang et al. 2023). Aperiodic activity can also confound detection of oscillatory brain activity (Donoghue, Dominguez, et al. 2020; Merkin et al. 2023). Thus, separating oscillatory from aperiodic activity is important for advancing our understanding of the relationship between brain dynamics, cognition, and behavior.

Electroencephalography (EEG) noninvasively captures brain electrical activity, and is often used for drawing inference about oscillations in the human brain. EEG studies often analyze post‐stimulus oscillatory power changes by transforming data to a time‐frequency representation, then applying a correction for signal power in some pre‐stimulus baseline period. This normalization is supposed to reveal brain stimulus‐locked oscillations. However, aperiodic power fluctuates during cognitive processing, including working memory (Preston et al. 2022) and attention (Kałamała et al. 2023). These fluctuations imply that correction using a pre‐stimulus period will not correctly model and remove stimulus‐locked aperiodic power changes. Baseline normalization can also return unstable results depending on varying levels of noise (Gyurkovics et al. 2021). We argue that existing methods for baseline correction of time‐frequency data do not fully correct for stimulus‐locked aperiodic power dynamics. On the other hand, explicit computational approaches for separating oscillatory and aperiodic power spectra have gained attention for EEG analysis. *Specparam* (Donoghue, Haller, et al. 2020) separates aperiodic and oscillatory contributions by fitting a line to the log‐transformed power spectrum, then fitting Gaussians to the flattened (aperiodic removed) remaining power. This approach is applied to frequency‐domain power spectra, ignoring time‐varying dynamics of the signal. Wilson et al. (2022) report the extension of specparam to time‐frequency data, calling the resulting approach Spectral Parameterization Resolved in Time (SPRiNT). Approaches like SPRiNT enable time‐resolved parameterization of EEG oscillatory and aperiodic power, allowing measurement of their respective contributions to EEG dynamics during cognition.

In this registered report, we applied time‐resolved spectral parameterization to EEG recordings during cognitive tasks that tap cognitive control and reinforcement processing. Neural dynamics recorded during many cognitive paradigms show similar patterns, including synchronization of centro‐parietal delta (~1–3 Hz) and mediofrontal theta (~4–8 Hz) and corresponding desynchronization of posterior alpha (~9–13 Hz) and central beta (~15–30 Hz) (Başar et al. 2001; Klimesch 1999). These dynamics are observed during cognitive control (Eisma et al. 2021; Rawls, Miskovic, and Lamm 2020), reinforcement processing (Bernat et al. 2015; Cavanagh 2015; Hager et al. 2022; Rawls, Miskovic, Moody, et al. 2020), and other EEG paradigms, including oddball detection (Bernat et al. 2007) and working memory (Jensen and Tesche 2002). Despite the general nature of these brain dynamics, the respective contributions of oscillatory and aperiodic activity to these signatures of cognition are not established. As far as we are aware, this is the first published report on the parameterization of dynamic EEG time‐frequency characteristics recorded during cognitive tasks into separate aperiodic and oscillatory components. Briefly, our study demonstrates the essentially oscillatory nature of EEG task time‐frequency metrics while also indicating unexpected aperiodic signal dynamics.

## 2 Methods

### 2.1 Participants

The sample consisted of US military veterans who had been deployed to Operations Iraqi Freedom or Enduring Freedom. Recruitment targeted veterans with likely posttraumatic stress disorder (PTSD) diagnoses as well as non‐treatment‐seeking veterans with similar deployment experiences. Study procedures were approved by the Institutional Review Boards at the Minneapolis Veterans Affairs Health Care System and the University of Minnesota, and study participants completed a written informed consent process prior to undergoing the study procedures. For a detailed description of the two samples, see Davenport et al. (2014) and Davenport et al. (2016). Here, we included only those participants who were not diagnosed with PTSD or mTBI following deployment (flanker *n* = 61, gambling *n* = 46). During data preparation, we discovered three additional usable flanker datasets, and conversely, we were unable to process two gambling datasets, modifying the final sample sizes to *n* = 64 (mean age = 34.78 ± 8.98, range = 23–62) and *n* = 44 (mean age = 32.45 ± 8.68, range = 22–54), respectively.

### 2.2 Cognitive Paradigms

We analyzed EEG data recorded during two paradigms that recruit common EEG signatures that are assumed to be oscillatory. The first was an Eriksen Flanker task (Eriksen and Eriksen 1974) that presented congruent (<<<<< or >>>>>) or incongruent (<<><< or >><>>) sets of arrows (50% incongruent). Participants were instructed to respond with their left thumb if the middle arrow pointed left, and to respond with their right thumb if the middle arrow pointed right, while ignoring the distractor arrows on either side of the target arrow. Participants completed 400 trials, which were divided into four blocks with self‐paced breaks in between. The task required approximately 20 min to complete. The main behavioral outcomes were mean response times and accuracy rates, calculated separately for congruent and incongruent trials.

**FIGURE 1:**
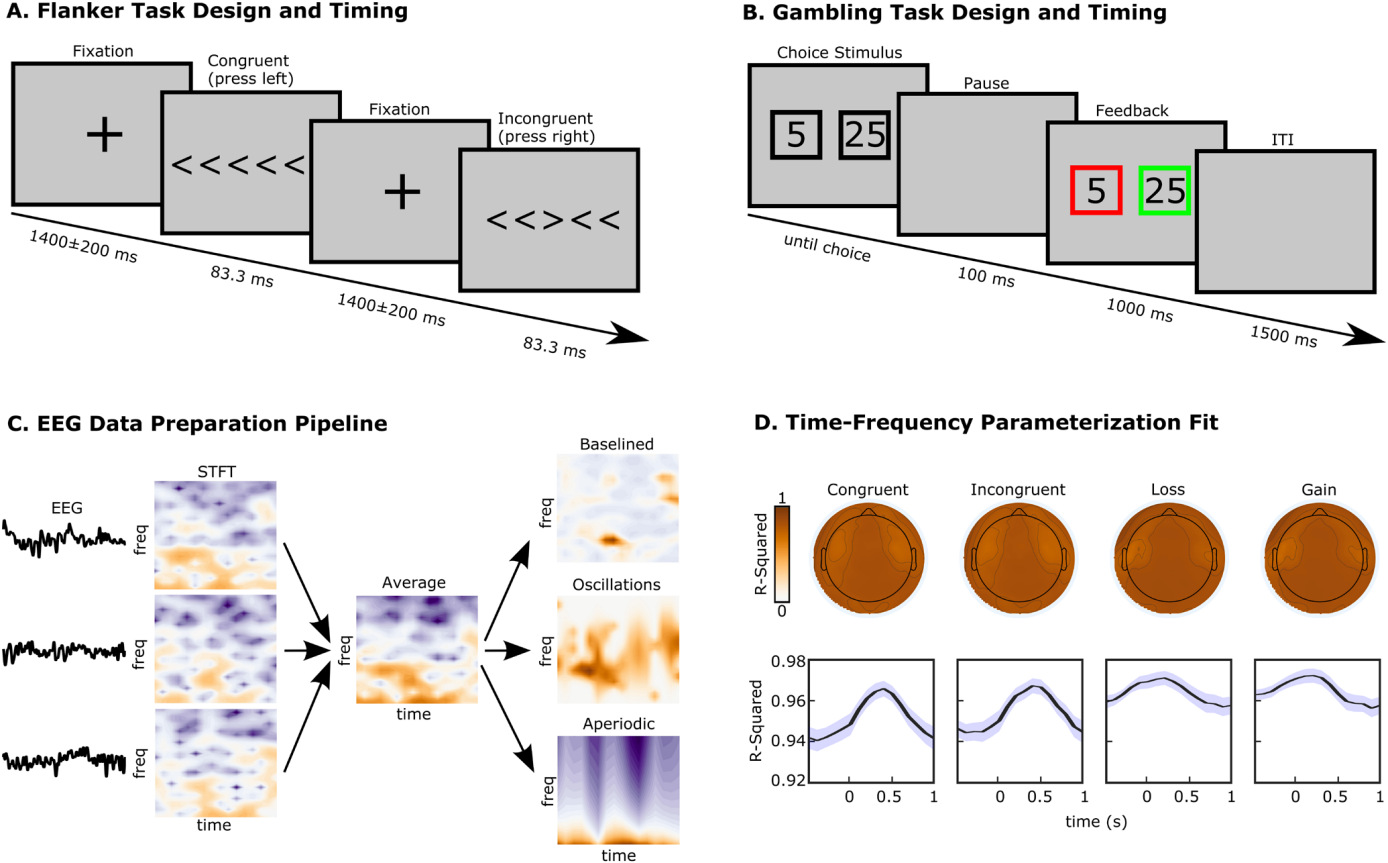
Task design for the two tasks analyzed here, and overall EEG analysis strategy. (A) Design and timing of the flanker task. (B) Design and timing of the gambling task. (C) Analysis and parameterization pipeline for task EEG data. Single trials of data were TF‐transformed, followed by averaging. From trial averages, we extracted estimates of total, baselined, oscillatory, and aperiodic time‐varying power for further analysis. (D) Time‐Frequency parameterization goodness‐of‐fit, shown topographically and as time series. Shading around time series represents SEM.

The second paradigm was a gambling paradigm (Gehring and Willoughby 2002). Each trial offered participants a two‐option forced choice. Options were 5 or 25 points, and could be paired in any fashion (i.e., 5/5, 5/25, 25/5, or 25/25). All four pairings were presented equiprobably. Choices were presented within black squares which remained on the screen until participants selected one option. One hundred ms following the choice, each square turned red or green. If the chosen option turned green, the indicated amount was added to the participant's running score. If the chosen option turned red, the indicated amount was instead subtracted from the participant's running score. The color of the unchosen option also changed, to indicate what the outcome would have been if the participant had instead chosen that option. Participants completed 256 trials, divided into eight blocks with self‐paced breaks in between. This task required approximately 20 min to complete. The primary behavioral outcome was risky choice proportion, defined as the percentage of times a participant chose the “25” option when presented with a choice between “5” and “25.” This risky choice proportion was calculated separately for trials following gains and losses. Diagrams of the two tasks are shown in Figure 1.

### 2.3 EEG Acquisition and Preprocessing

EEG were sampled at 1024 Hz using a 128‐channel BioSemi ActiveTwo EEG system, acquired reference‐free (via CMS/DRL sensors). EEG data from both samples were preprocessed using automated methods. Data were high‐pass filtered (1 Hz; down 6 dB/octave; 8 voices/octave), low‐pass filtered (30 Hz; down 6 dB/octave; 8 voices/octave), and resampled to 250 Hz. We detected, removed, and spherically interpolated (Perrin et al. 1989) bad channels using the cleanrawdata plugin for EEGLAB (Kothe and Makeig 2013). Single trials for the flanker paradigm were epoched surrounding congruent and incongruent stimuli, and single trials for the gambling paradigm were epoched surrounding gain and loss feedback. Epochs extended from −750 to +1250 ms surrounding events of interest to allow for STFT windowing (1/2 of the 500 ms window is removed from each side) (Cohen 2014; Keil et al. 2022). Trials containing extreme artifacts were rejected using a threshold of 500 μV. Temporal ICA was computed (Makeig et al. 1996), followed by detection and removal of artifact components (eye, cardiac, muscle, or line noise‐related) using ICLabel (Pion‐Tonachini et al. 2019) with a confidence threshold of 70%. Remaining artifacts were removed via thresholding single trials at 125 microvolts.

### 2.4 Time‐Frequency Transformation

The SPRiNT approach for parameterizing TF surfaces is more accurate for short‐time Fourier transforms (STFTs) than for wavelets (Wilson et al. 2022). Thus, we calculated spectrograms from single‐trial data recorded during the flanker and gambling paradigms. Transforms utilized 500 ms (125 sample) Hann‐windowed segments with 80% overlap. This window provided a Rayleigh frequency (low‐frequency resolution) of 2 Hz, and we zero‐padded FFTs to achieve a minimum frequency of 1 Hz, ensuring we can resolve oscillations of 2 Hz or above (i.e., into delta ranges) with the chosen STFT parameters. This analysis provided a TF representation with frequencies ranging from 1 to 30 Hz in steps of 1 Hz and times ranging from −500 to +1000 ms in 100 ms steps.

Since evoked, or phase‐locked, power increases can create the false appearance of oscillations, for each frequency, we removed the phase‐locked power by reconstituting the complex signal from the phase and power components, averaging over trials, and subtracting the average from single trials, followed by re‐extracting power and phase. We verified the success of this removal by inspecting intertrial phase coherence maps, which predictably showed no evidence of phase locking following ERP subtraction. That is, prior to ERP removal, averaged ITC values (across subjects) reached normal values during ERP peaks (as high as 0.4–0.5). After ERP removal, averaged ITC values (across subjects) never surpassed ~0.04. We note that ERP subtraction in single trials, while the de facto method for removal of evoked power, is not a foolproof method and can even introduce spurious effects like edge artifacts on occasion. As such, in the supplement we replicate all key analyses using total power without the ERP subtracted. All key effects were equivalent across the two analyses. Following removal of evoked power, we averaged single‐trial TF power within each condition of interest prior to parameterization to increase signal‐to‐noise ratio.

For certain correlation analyses, we also computed baseline‐corrected power using percent change from baseline. This choice was made during preregistration because it is more robust than dB when baseline power is very low. Note that baseline‐corrected power was never parameterized, and these power values were used only for correlation analyses.

### 2.5 Time‐Frequency Spectral Parameterization

For time‐resolved spectral parameterization, we used default parameters from specparam (peak widths = [0.5 12], peak detection threshold = 2.0 SD, no max number of peaks, no minimum peak height, aperiodic mode = fixed). As a metric of parameterization quality, we calculated mean *R* ^2^ for model fits. Note that the study did not use a priori frequency bands, instead relying on testing at every frequency point followed by cluster correction (see below). Specparam provides an excellent accounting for neural power spectra, and, as with all models, has the potential to overfit the data. Due to the large volume of data produced, we manually examined the spectral parameterization results of ~10% of the subjects from each sample to check for overfitting. We do note that since our primary analyses apply to condition differences rather than the raw output of specparam, any overfitting would not be expected to influence results since the overfitting would equally influence both conditions. Note further that the SPRiNT algorithm for continuous data, as published by Wilson et al. (2022) clustered peaks over three‐second windows to reduce false positives; this clustering is inappropriate for event‐related data due to the expectation of immediate stimulus‐related oscillatory changes. As such, we did not use the temporal clustering step of SPRiNT, rendering our approach equivalent to running specparam on every STFT‐transformed temporal data sample. The time‐frequency analysis pipeline is illustrated in Figure 1C.

### 2.6 Power Analysis

Since data were previously collected, the power analysis is post hoc. However, the power analysis was completed during Stage 1 review before data were analyzed. We determined that for a paired‐sample *t*‐test, our smaller sample of *n* = 44 achieves a power of 0.8 at an alpha of 0.05 with a medium effect size of *d* = 0.43, and likewise, for a correlation test, our smaller sample achieves a power of 0.8 for a medium effect size of *r* = 0.4. A recent meta‐analysis of the effect of cognitive working load on frequency‐domain EEG (Chikhi et al. 2022) showed medium‐large effect sizes (Hedge's *g*) for the effect of cognitive working load on theta (*g* = 0.68), alpha (*g* = −0.25), and beta (*g* = 0.5) power, including several studies using the STFT time‐frequency method, such as that used here. Another meta‐analysis of theta‐band event‐related potentials noted a medium‐large association between frontal theta and post‐loss switching (*z* = 0.55) albeit a somewhat smaller association between theta and post‐response slowing (*z* = 0.2). As such, our sample sizes are appropriate for the proposed analyses.

### 2.7 Overall Statistical Approach

We analyzed full‐scalp TF condition differences, and TF associations with task performance, using two‐sided threshold‐free cluster enhancement (TFCE) tests (Mensen and Khatami 2013) implemented in the ept‐tfce software. This test uses permutation‐based threshold‐free cluster correction for multiple comparisons, and in all cases will be evaluated using a significance cutoff of tfce‐corrected *p* < 0.05. Tests were computed for every channel, frequency, and time point rather than relying on a priori average frequency ranges.

### 2.8 Tests for Condition Differences in Parameterized Power

We assessed for congruent‐incongruent differences (flanker), and for gain‐loss differences (within each level of low and high point outcome) and low‐high point outcome differences (within each level of gain and loss) (gambling), at each sensor‐time‐frequency sample. We anticipated that these tests would reveal (1) significant increases in mediofrontal theta oscillatory power for incongruent trials (compared with congruent) and for loss trials (compared with gain), and (2) significant increases in central‐parietal delta oscillatory power for congruent trials (compared with incongruent), for gain trials (compared with loss), and for high outcomes (compared with low). On the other hand, we anticipated observing alpha and beta oscillations during baseline periods, which would be suppressed during post‐stimulus periods, but we did not hypothesize observing any condition differences in these oscillatory bands. We did not have distinct hypotheses for our analyses of aperiodic spectra condition differences, given the novelty of this analysis. This analysis was used to provide evidence for or against the common claim that baseline‐corrected total power as measured during cognitive tasks primarily measures oscillatory power changes.

### 2.9 Correlations Between Total and Parameterized Power

We tested correlations between total power and (1) aperiodic and (2) oscillatory power, for data that were baseline‐corrected (percent change) and for data that were not baseline‐corrected. We calculated these correlations across subjects for each condition, each sensor, and each time‐frequency point. The significance of correlations was assessed by correcting the resulting *p* values within each condition using TFCE. This permutation‐based significance test circumvents typical assumptions of normality. In this permutation framework, *p* values are derived by comparing the observed correlation against an empirical null distribution created from randomly shuffled data; thus, deviations from normality or outliers do not impact correlation results as they do in a parametric analysis. We anticipated that this analysis would show that (1) aperiodic power is highly correlated with non‐baseline‐corrected power, whereas (2) oscillatory power is highly correlated with baseline‐corrected power. This analysis was used to provide evidence for or against the common claim that baseline correction provides a simple method for 1/f correction in TF analysis.

### 2.10 Correlations Between Parameterized Power and Behavior

We tested whether oscillatory and aperiodic task TF power were associated with behavior between subjects. Separately for oscillatory and aperiodic power estimates and for congruent and incongruent flanker trials, we correlated TF power at each sensor‐time‐frequency point with mean RTs and accuracy rates. Separately for oscillatory and aperiodic power estimates and for gain and loss gambling trials, we correlated TF power at each sensor‐time‐frequency point with risky choice proportion. For flanker analyses, we predicted that mediofrontal theta oscillations and central‐parietal delta oscillations would be negatively associated with RTs across subjects, whereas posterior alpha oscillations would be positively correlated with RTs across subjects. For gambling task analyses, we predicted that central‐parietal delta oscillations would be negatively associated with risky choice proportion following wins. This analysis was used to provide evidence for or against the common claim that post‐stimulus oscillatory changes are the primary contributor to EEG‐behavior associations. Significance of correlations was again assessed using a permutation test that does not assume normality.

### 2.11 Statistical Results Plotting and Reporting

For all results, we report statistical results as cluster maximums within specific frequency bands, where delta = 1–3 Hz, theta = 4–8 Hz, alpha = 9–14 Hz, and beta = 15–30 Hz. Note that this does not necessarily properly represent the nature of TFCE correction, as clusters can extend across multiple frequency bands. Still, these frequency bands are commonly used for interpretational purposes, and so we split our reporting across bands to better place our results within the existing literature. We thus plot statistics averaged within frequency and time windows for viewing and interpretation ease. For every test, we plot a series of topographic plots, with *t* (or *r*)‐statistics shown masked by significance. Topographic plots are shown using time bins extending from −400 ms before stimulus to 800 ms after stimulus. For brevity, and because of the large number of plots shown, we show only topos with 400 ms spacing. Within each frequency bin for plotting, a time‐frequency surface from the channel with the greatest cluster statistic is shown, with contours outlining significant effects. The selected sensor is denoted with a cyan circle. For each frequency bin, the portion of the surface that is not in the bin is covered by transparent gray squares to highlight the important portions of the surface. Cyan lines on surfaces denote the time‐varying activity or statistic (averaged over individual frequencies) within the frequency bin of interest.

### 2.12 Summary of Analyses

Overall, our analyses are meant to provide the first empirical tests to validate the common claim that essential EEG task time‐frequency metrics, specifically mediofrontal theta synchronization, parietal delta synchronization, posterior alpha desynchronization, and central beta desynchronization, are manifestations of changes in brain oscillations rather than changes in aperiodic noise. We systematically test the common claims that oscillations underlie baseline‐corrected task condition differences, that oscillations can be revealed by baseline correction, and that oscillations underlie common EEG‐behavior associations.

## 3 Results

### 3.1 EEG Time‐Frequency Spectral Parameterization Reveals the Oscillatory Nature of Common Task‐Evoked Features

**FIGURE 2:**
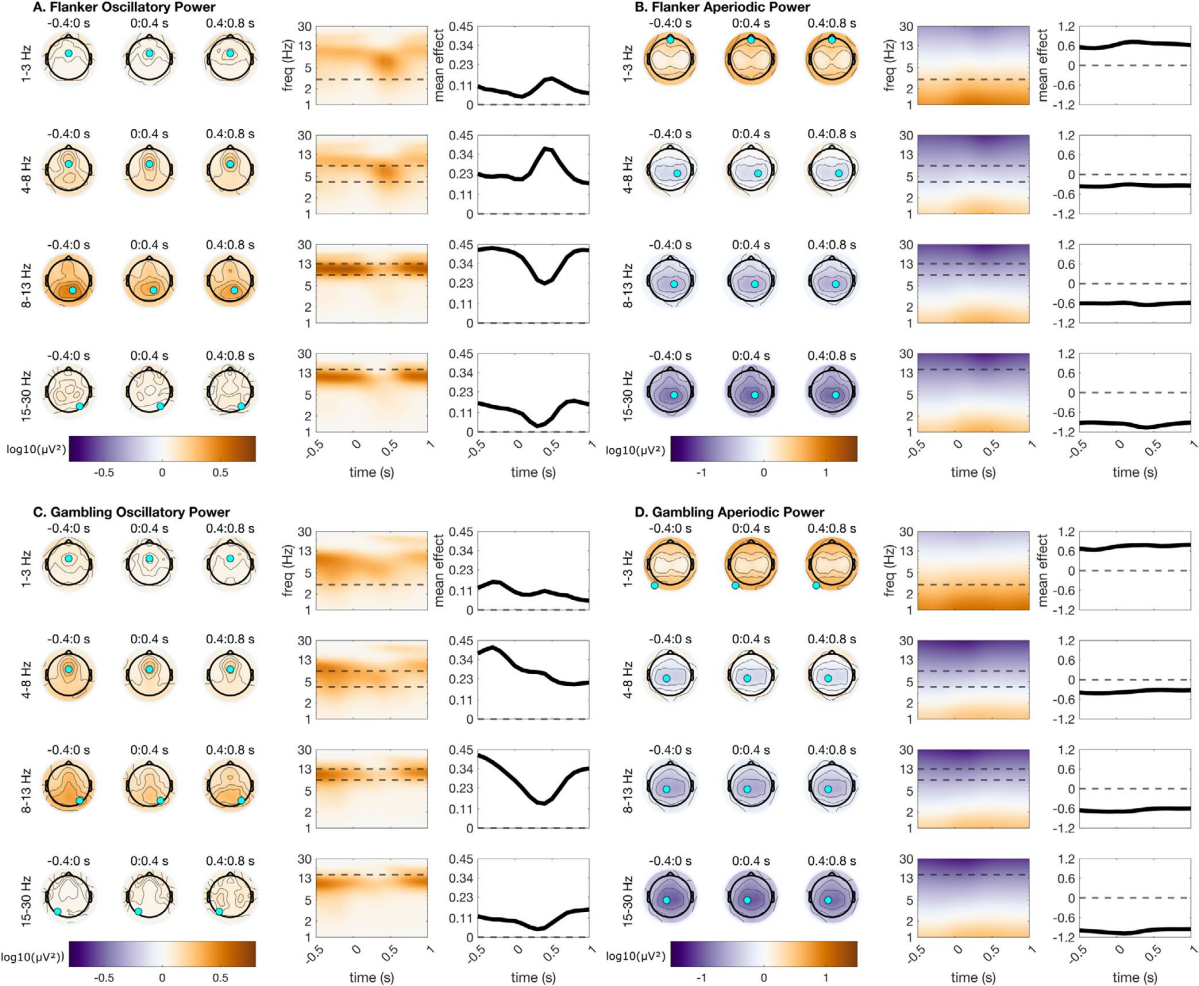
Time‐frequency parameterization of condition‐averaged activity from the Flanker (stimulus‐locked) and gambling paradigms (feedback‐locked). Topographic plots are shown using time bins extending from −400ms before stimulus to 800 ms after stimulus. Within each frequency bin for plotting, a time‐frequency surface from the channel with the greatest measurement is shown (log‐spaced frequency axis). The selected sensor is denoted with a cyan circle. All topographic plots are divided by frequency bin, where delta = 1–3 Hz, theta = 4–8 Hz, alpha = 9–14 Hz, and beta = 15–30 Hz. (A) Oscillatory power for flanker. (B) Aperiodic power for flanker. (C) Oscillatory power for gambling. (D) Aperiodic power for gambling.

Time‐frequency parameterization was high quality for both datasets (average *R* ^2^ = 0.98). Applying spectral parameterization to condition‐averaged (but not baseline‐corrected) EEG task time‐frequency maps revealed several expected patterns (see Figure 2). Posterior alpha oscillations were high at rest, faded during stimulus processing, and reappeared post‐stimulus. Conversely, mediofrontal theta oscillations were apparent during stimulus processing more so than baseline periods. Beta oscillations were also observed during baseline periods over central and posterior regions. Similar to alpha oscillations, these beta oscillations faded during stimulus processing and reappeared post‐stimulus. We also observed post‐stimulus oscillations in delta frequencies, although note that our parameterization method could not resolve oscillations below 2 Hz. For condition‐averaged plots of parameterized oscillations in the flanker task, see Figure 2A, and for the gambling task, see Figure 2C.

Meanwhile, aperiodic power was high during both baseline periods and stimulus processing periods, but appeared to increase at low frequencies and decrease at high frequencies during stimulus processing. For condition‐averaged plots of parameterized aperiodic in the flanker task, see Figure 2B, and for the gambling task see Figure 2D. Our primary conclusion is that the removal of phase‐locked (evoked) activity, followed by TF parameterization, reveals that the most frequently reported induced task time‐frequency features appear to be oscillatory, but the aperiodic portion of activity also shows stimulus‐ and feedback‐locked dynamics.

### 3.2 EEG Task Oscillatory Time‐Frequency Condition Differences

When we tested for condition differences in the conflict monitoring and gambling datasets, we were interested in observing several commonly observed features: mediofrontal theta should increase for incongruent trials compared with congruent (conflict monitoring), and for losses compared with gains (gambling). Meanwhile, we would expect posterior alpha to desynchronize more greatly for incongruent than congruent trials, whereas feedback differences in alpha are less commonly observed. Gains, compared with losses, are associated with increased post‐stimulus delta power. Meanwhile, congruent trials are often associated with higher delta than incongruent trials earlier in the trial, while this difference shifts to higher delta for incongruent trials later in the trial. This difference in timing of delta could potentially be mostly attributed to P3 amplitude effects in typical TF studies, but here we subtract the ERP from every single trial, so this is not the case for our results. Beta activity is generally suppressed for incongruent, compared with congruent trials, but is not responsive to gains vs. losses.

**FIGURE 3:**
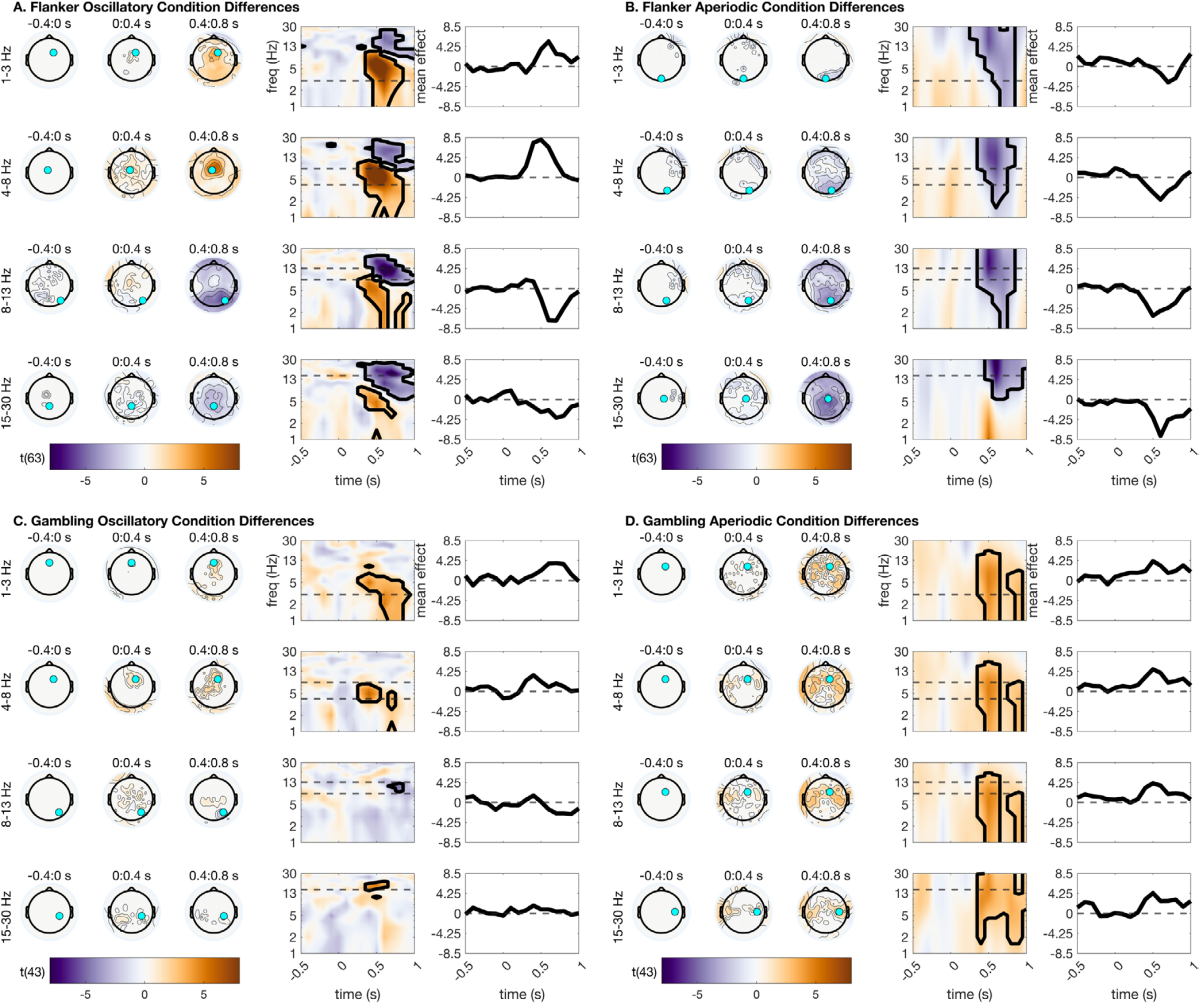
Parameterized time‐frequency difference plots from the Flanker and gambling paradigms. Topographic plots are shown using time bins extending from −400 ms before stimulus to 800 ms after stimulus. Within each frequency bin for plotting, a time‐frequency surface from the channel with the greatest cluster statistic is shown (log‐spaced frequency axis). The selected sensor is denoted with a cyan circle. Contours on topos and on TF surfaces highlight significant clusters; topos are additionally significance‐masked for readability. All topographic plots are divided by frequency bin, where delta = 1–3 Hz, theta = 4–8 Hz, alpha = 9–14 Hz, and beta = 15–30 Hz. Flanker plots show incongruent‐congruent, and gambling plots show loss‐gain. Topographic plots are masked by significance, so any contours outline significant effects. Likewise, contours on TF surfaces highlight significant clusters. (A) Oscillatory condition differences for flanker. (B) Aperiodic condition differences for flanker. (C) Oscillatory condition differences for gambling. (D) Aperiodic condition differences for gambling.

Our analysis revealed that the majority of these commonly observed EEG condition differences in cognitive control and reinforcement processing can be attributed to oscillations, rather than aperiodic power changes. For incongruent trials compared with congruent trials in the Flanker task, we observed prominent increases in parameterized mediofrontal theta oscillations, *t*(63) = 12.17, *p* < 0.001, and corresponding decreases in parameterized posterior alpha oscillations, *t*(63) = −9.27, *p* < 0.001 (Figure 3A). We also observed frontal/central delta oscillation increases, *t*(63) = 7.76, *p* < 0.001, and beta oscillation decreases, *t*(63) = −8.78, *p* < 0.001, for incongruent trials. For the gambling task, we observed prominent loss‐related increases in mediofrontal theta oscillations, *t*(43) = 5.81, *p* < 0.001, frontal/central delta oscillations, *t*(43) = 5.11, *p* = 0.002, and in lateralized beta oscillations, *t*(43) = 5.29, *p* = 0.002 (Figure 3C). From this analysis, we can conclude that many of the typical EEG task time‐frequency condition differences are essentially oscillatory in nature and are recapitulated in parameterized time‐frequency oscillations.

### 3.3 EEG Task Aperiodic Time‐Frequency Condition Differences

On the other hand, when we tested for aperiodic condition differences, we had few hypotheses due to the novel nature of this analysis. One hypothesis was that during stimulus processing, we anticipated that low‐frequency aperiodic power would increase and high‐frequency aperiodic power would decrease, in line with prior evidence that the slope of the power spectrum becomes less steep during attentional processing. However, we had no hypotheses about the direction of this effect.

For the flanker task, our analysis revealed a large cluster of broadband aperiodic differences over central and parietal sensors, extending from delta to beta frequencies and active across a wide time period during stimulus processing, *t*(63) = −8.26, *p* < 0.001 (Figure 3B). This indicates that congruent trials are associated with enhanced broadband aperiodic power over central and parietal sensors. Intriguingly, in the gambling task, a large central cluster of sensors also showed broadband aperiodic power differences, *t*(63) = 4.91, *p* = 0.001 (Figure 3D). However, in this case, loss trials instead showed enhanced broadband aperiodic power, extending from delta to beta frequencies. This provides the first evidence of large‐scale aperiodic shifts during both cognitive control and feedback processing, implying that congruent trials and loss processing are both associated with enhanced broadband aperiodic power over central and lateralized sensors.

### 3.4 EEG Time‐Frequency Correlations: Parameterized and Total Power

**FIGURE 4:**
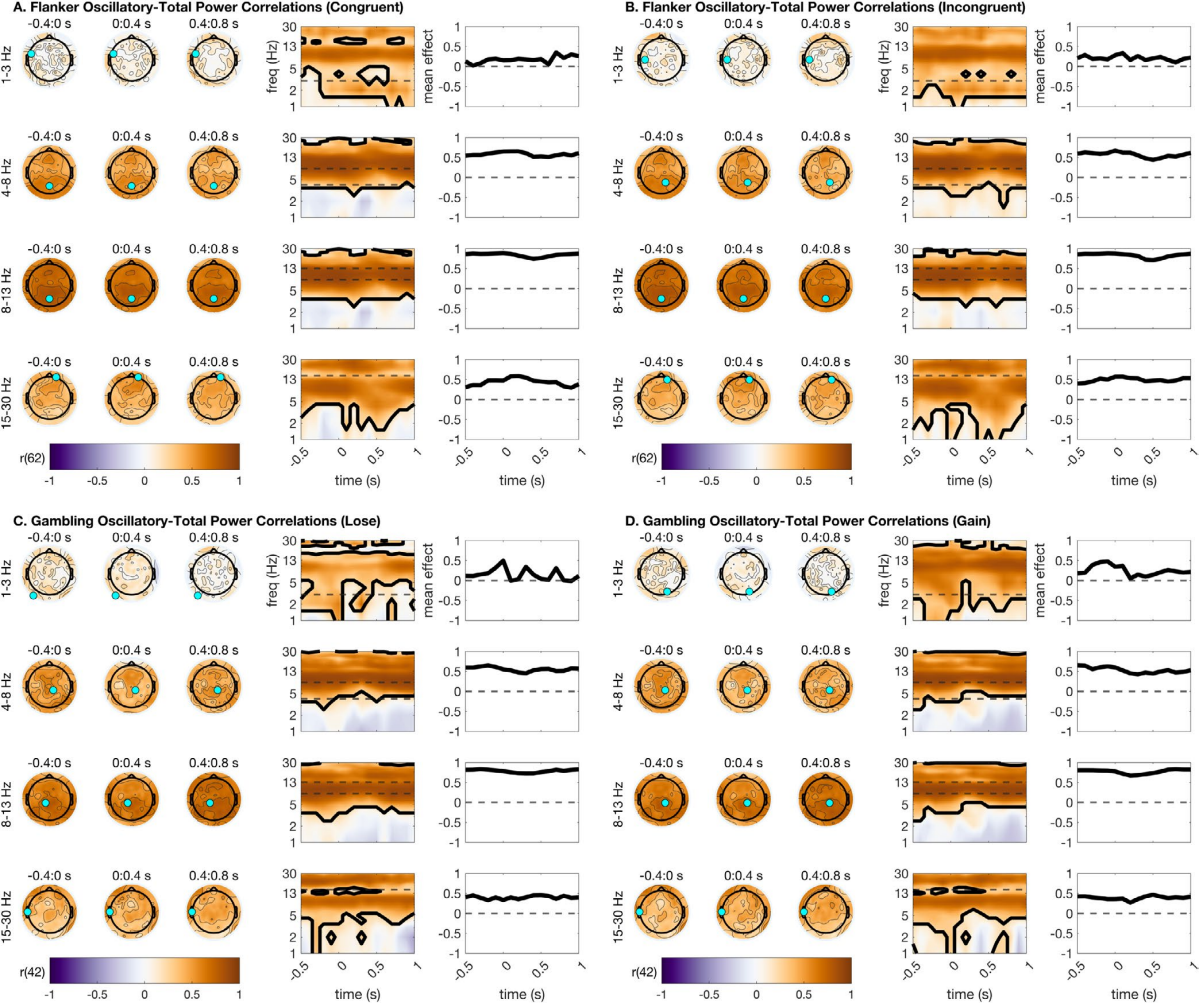
Extent to which total power estimates correlate with parameterized oscillation estimates. This indicates frequencies that are essentially oscillatory prior to baseline correction. Topographic plots are shown using time bins extending from −400 ms before stimulus to 800 ms after stimulus. Within each frequency bin for plotting, a time‐frequency surface from the channel with the greatest cluster statistic is shown (log‐spaced frequency axis). The selected sensor is denoted with a cyan circle. Contours on topos and on TF surfaces highlight significant clusters; topos are additionally significance‐masked for readability. All topographic plots are divided by frequency bin, where delta = 1–3 Hz, theta = 4–8 Hz, alpha = 9–14 Hz, and beta = 15–30 Hz. Correlations were high across alpha, and to a lesser extent theta and beta, bands. (A) Total power–oscillatory correlations for the flanker task, congruent trials. (B) Total power–oscillatory correlations for the flanker task, incongruent trials. (C) Total power–oscillatory correlations for the gambling task, loss trials. (D) Total power–oscillatory correlations for the gambling task, gain trials.

EEG is dominated by aperiodic activity (with the sole exception of the alpha rhythm, which can be a stronger contributor to the EEG than the aperiodic signal in some cases). Baseline correction is argued to reveal time‐locked oscillations in the EEG and remove aperiodic power contributions. If this is true, then parameterized aperiodic power should correlate highly with EEG that has not been baseline‐corrected, and parameterized oscillatory power should correlate highly with EEG that has been baseline‐corrected. To test this hypothesis, we correlated the parameterized oscillatory and aperiodic data with total TF. We first completed this test for total power estimates with no baseline correction applied. We anticipated that correlations between oscillations and total power would be greatest in frequencies that we have previously shown to be oscillatory (especially theta and alpha). These hypotheses were upheld across all task conditions (Figure 4). Total power–oscillation correlations exceeded 0.8 (*p* < 0.001) in clusters focused around mediofrontal theta, parietal alpha, and central beta. These correlations were much weaker and generally non‐significant for delta ranges. This supports the claim that time‐locked activity in theta and especially alpha frequencies contains high information about oscillations even before baseline correction. See Figure 4 for a full summary of all statistical results from this analysis.

**FIGURE 5:**
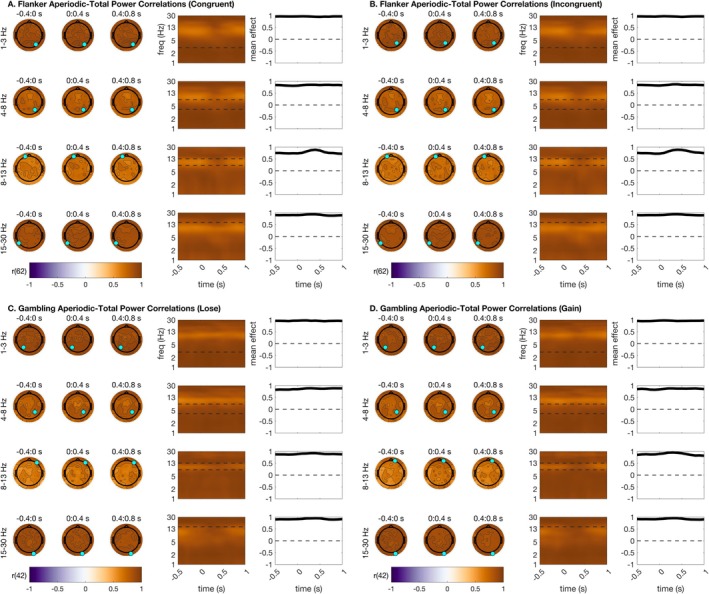
Total power correlations with parameterized aperiodic estimates. Correlations were uniformly extremely high. Topographic plots are shown using time bins extending from −400 ms before stimulus to 800 ms after stimulus. Within each frequency bin for plotting, a time‐frequency surface from the channel with the greatest cluster statistic is shown (log‐spaced frequency axis). The selected sensor is denoted with a cyan circle. Contours on topos and on TF surfaces highlight significant clusters; topos are additionally significance‐masked for readability. All topographic plots are divided by frequency bin, where delta = 1–3 Hz, theta = 4–8 Hz, alpha = 9–14 Hz, and beta = 15–30 Hz. (A) Total power‐aperiodic correlations for the flanker task, congruent trials. Note that all points are significant so no masking is needed. (B) Total power‐aperiodic correlations for the flanker task, incongruent trials. (C) Total power‐aperiodic correlations for the gambling task, loss trials. (D) Total power‐aperiodic correlations for the gambling task, gain trials.

We anticipated that the comparison of total power to aperiodic power, meanwhile, would reveal high correlations between total power without baseline correction and aperiodic power, across almost all frequencies except for highly oscillatory frequencies like alpha and to a lesser extent theta. This hypothesis was upheld, as total power–aperiodic correlations exceeded 0.9 (*p* < 0.001) across almost the entire scalp (Figure 5), in line with observations that the EEG signal is dominated by aperiodic activity (except for the posterior alpha oscillation). See Figure 5 for a full summary of all statistical results from this analysis.

We additionally implemented a Fisher's *z*‐test to assess for condition differences in correlation magnitude between oscillatory and aperiodic correlation surfaces in each condition. This analysis clarified whether oscillations or aperiodic power correlated more highly with total power. As expected, total power correlated more highly with aperiodic power than with oscillatory power in all topographic distributions and in all frequency ranges except for parietal and mediofrontal alpha. Parietal alpha total power–oscillatory correlations modestly exceeded total power‐aperiodic correlations. See supplemental materials for plots of Fisher z‐tests for correlation differences (Figures S9 and S10).

### 3.5 EEG Time‐Frequency Correlations: Parameterized and Baselined Power

We next asked whether the parameterized oscillatory and aperiodic data correlated highly with distinct portions of the baselined TF. This analysis is largely in response to the common claim that baseline correction can remove the influence of signal aperiodics for task EEG (Cohen 2014; Gyurkovics et al. 2021). We anticipated that this analysis would reveal high correlations between parameterized oscillations and baseline‐corrected power, but not equally across all frequencies. Specifically, we hypothesized based on the literature that mediofrontal theta would correlate highly between oscillatory and baseline‐corrected surfaces, since our prior analyses indicated that parameterized oscillations preserve classic mediofrontal theta condition differences (Figure 3).

**FIGURE 6:**
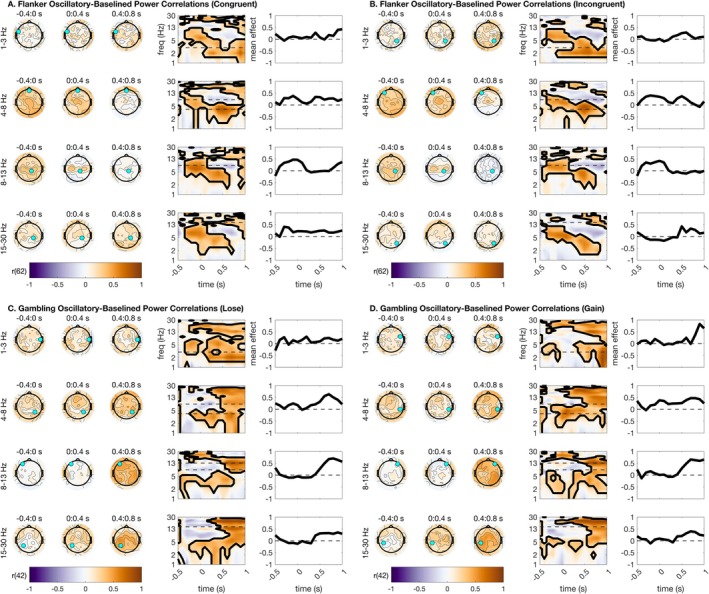
Baselined power correlations with parameterized oscillation estimates. Correlations were high across theta, alpha, and beta ranges. Topographic plots are shown using time bins extending from −400 ms before stimulus to 800 ms after stimulus. Within each frequency bin for plotting, a time‐frequency surface from the channel with the greatest cluster statistic is shown (log‐spaced frequency axis). The selected sensor is denoted with a cyan circle. Contours on topos and on TF surfaces highlight significant clusters; topos are additionally significance‐masked for readability. All topographic plots are divided by frequency bin, where delta = 1–3 Hz, theta = 4–8 Hz, alpha = 9–14 Hz, and beta = 15–30 Hz. (A) Baselined power–oscillatory correlations for the flanker task, congruent trials. (B) Baselined power–oscillatory correlations for the flanker task, incongruent trials. (C) Baselined power–oscillatory correlations for the gambling task, loss trials. (D) Baselined power–oscillatory correlations for the gambling task, gain trials.

We first report correlations of baseline‐corrected power estimates with parameterized oscillations. We found that our hypotheses were largely true (Figure 6), as mediofrontal theta correlations were significant across all task conditions. This suggests that baseline‐corrected frontal theta activation reflects aspects of oscillatory activity. Uniformly, lateralized beta also correlated highly between oscillatory and baseline‐corrected surfaces. As such, baseline correction for beta frequencies appears to produce a signal that meaningfully approximates the oscillations in the beta band. Intriguingly, correlations were far less distinct for parietal alpha, implying that baseline‐corrected alpha might provide a relatively poor estimate of alpha oscillations during cognitive tasks. See Figure 6 for a full summary of all statistical results from this analysis.

**FIGURE 7:**
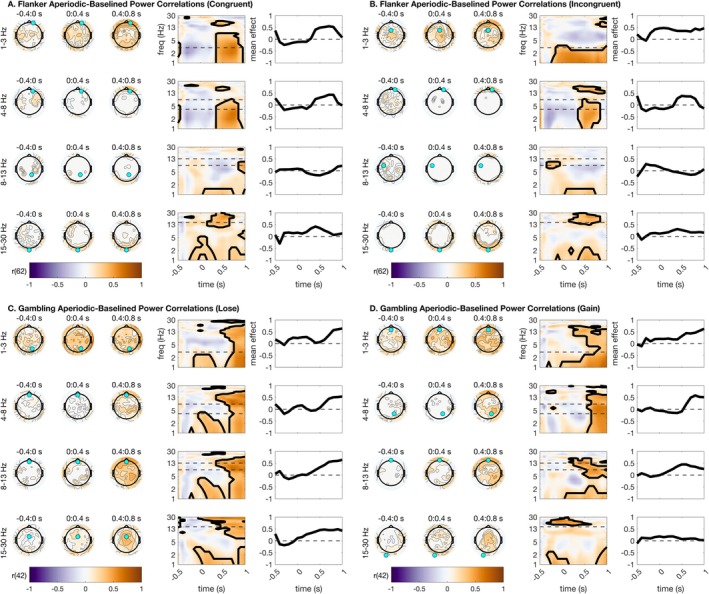
Baselined power correlations with parameterized aperiodic estimates. Correlations were high across delta, alpha, and beta ranges. Topographic plots are shown using time bins extending from −400 ms before stimulus to 800 ms after stimulus. Within each frequency bin for plotting, a time‐frequency surface from the channel with the greatest cluster statistic is shown (log‐spaced frequency axis). The selected sensor is denoted with a cyan circle. Contours on topos and on TF surfaces highlight significant clusters; topos are additionally significance‐masked for readability. All topographic plots are divided by frequency bin, where delta = 1–3 Hz, theta = 4–8 Hz, alpha = 9–14 Hz, and beta = 15–30 Hz. (A) Baselined power‐aperiodic correlations for the flanker task, congruent trials. (B) Baselined power‐aperiodic correlations for the flanker task, incongruent trials. (C) Baselined power‐aperiodic correlations for the gambling task, loss trials. (D) Baselined power‐aperiodic correlations for the gambling task, gain trials.

However, we note from our previous analyses that there are strong condition differences in aperiodic power during stimulus processing (Figure 3), implying that baseline correction cannot possibly remove all (dynamic) aperiodic information from the signal. In this section, we report correlations of baseline‐corrected total power with parameterized signal aperiodic power. We found that parameterized aperiodic power correlated highly with baseline‐corrected total power in several interesting patterns. First, these correlations were significant across all conditions for central‐parietal delta activity during stimulus processing, suggesting that some previously reported delta power differences might in fact correlate highly instead with signal aperiodic estimates. Second, these correlations were high for parietal alpha and beta in the flanker task, and with central alpha and beta during the gambling task. Together, these results suggest that baseline correction appears to adequately reflect oscillations in theta frequency ranges, but is less accurate in capturing oscillations free of aperiodic contamination in delta, alpha, and beta ranges. For all statistical results from this analysis, see Figure 7.

We additionally implemented a Fisher's z‐test to assess for condition differences in correlation magnitude between oscillatory and aperiodic correlation surfaces in each condition. This analysis clarified whether oscillations or aperiodic power correlated more highly with baseline‐corrected power. The most salient result is that mediofrontal theta parameterized oscillations correlate much more highly with baseline‐corrected mediofrontal theta power than do periodic power shifts. We observe that baseline‐corrected alpha and beta generally also correlate more highly with oscillatory than aperiodic power. Meanwhile, we observed that central delta power changes correlated more highly with aperiodic than oscillatory power. These topographic effects imply that mediofrontal theta, as previously studied in the literature via baseline correction, is primarily an oscillatory signal, while central delta might be aperiodic instead. See supplemental materials for plots of Fisher's *z*‐tests for correlation differences.

### 3.6 Correlations Between Behavior and Parameterized EEG

**FIGURE 8:**
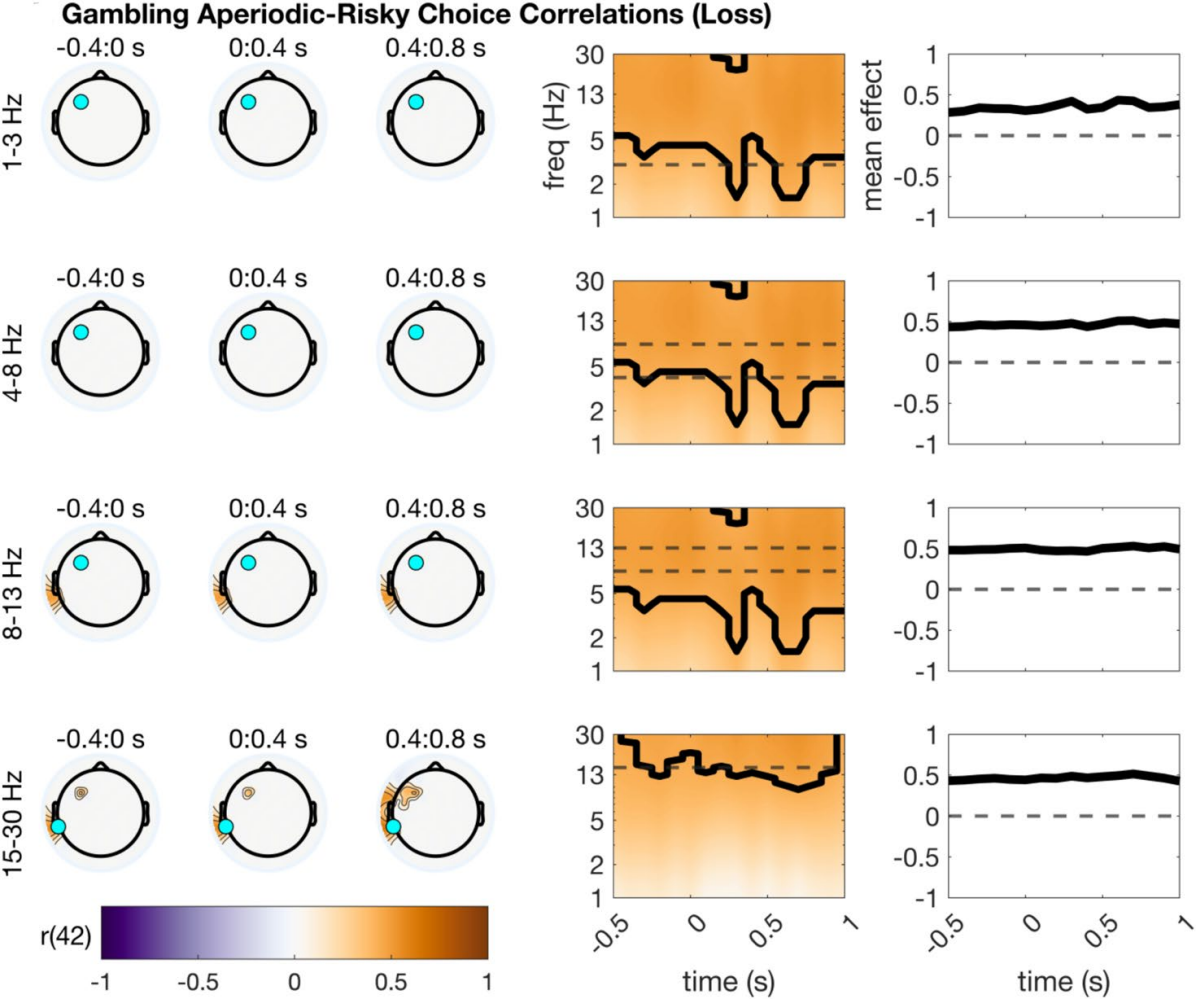
Correlations between parameterized gambling EEG and behavior. Topographic plots are shown using time bins extending from −400 ms before stimulus to 800 ms after stimulus. Within each frequency bin for plotting, a time‐frequency surface from the channel with the greatest cluster statistic is shown (log‐spaced frequency axis). The selected sensor is denoted with a cyan circle. Contours on topos and on TF surfaces highlight significant clusters; topos are additionally significance‐masked for readability. All topographic plots are divided by frequency bin, where delta = 1–3 Hz, theta = 4–8 Hz, alpha = 9–14 Hz, and beta = 15–30 Hz. Aperiodic‐risk correlation (loss) indicating that as aperiodic power increases across subjects, post‐outcome risky choice proportion also increases.

Finally, we asked whether the parameterized oscillatory and aperiodic data correlated highly with individual differences in behavior. Based on prior research, we anticipated that this analysis in the flanker task would reveal (1) negative correlations between frontal theta oscillations and RT, and (2) positive correlations between somatomotor beta oscillations and RT. Given previous interpretations of aperiodic power as primarily noise, we expected that higher aperiodic power would essentially be a proxy for noisier neural representations of task events. As such, we expected positive correlations between aperiodic power and RT, and negative correlations between aperiodic power and accuracy. For oscillatory power, none of the correlations were significant after TFCE correction (all *p* > 0.09). For the gambling task, based on prior evidence we likewise hypothesized that central delta might correspond to risky post‐win choice proportions across subjects. This hypothesis was not supported. Unexpectedly, we uncovered a statistically significant association between aperiodic power during loss processing and post‐loss risky choices, *r* = 0.52, *p* = 0.03. This effect appeared in a small central cluster of sensors and a small left‐lateralized frontal cluster of sensors, as well as a left‐lateralized temporal cluster of sensors. Interestingly, this suggests that there is some association between central‐frontal aperiodic activity and risky choices across subjects. For a summary of these results, see Figure 8.

## 4 Discussion

### 4.1 General Discussion

To separate the relative contributions of neural oscillations and aperiodic (1/f) signals captured in electrophysiological recordings, we report the application of modern computational techniques to explicitly model periodic and aperiodic contributions to time‐resolved EEG task responses (Donoghue, Haller, et al. 2020; Wilson et al. 2022). On the one hand, we confirm that mediofrontal theta synchronization during the need for cognitive control (Cavanagh et al. 2012; Cavanagh and Frank 2014; Eisma et al. 2021) and during loss processing (Bernat et al. 2015; Cavanagh and Shackman 2015; Shackman et al. 2011), and parietal alpha suppression during the need for cognitive control (Başar et al. 2001; Compton et al. 2011; Klimesch 1999), are entirely captured by modeled oscillations. On the other hand, we show previously unknown condition differences in aperiodic power during common tasks probing cognitive control (Eriksen and Eriksen 1974) and outcome processing (Gehring and Willoughby 2002). We interpret results in terms of the neural biophysical mechanisms giving rise to coordinated brain rhythms and aperiodic activity, and suggest that a more biophysically informed understanding of EEG is required to move past these issues.

### 4.2 Role of Oscillatory Activity During Cognitive Tasks

Parameterization of task time‐frequency activity revealed prominent induced mediofrontal theta and parietal alpha oscillations. In line with hypotheses and prior work, theta increased during stimulus processing (Cavanagh et al. 2012; Cavanagh and Frank 2014; Eisma et al. 2021), whereas alpha decreased during stimulus processing. This suggests that commonly observed task differences in theta and alpha bands do in fact reflect modulation of EEG oscillations, rather than confounding influences of aperiodic activity. Compatible interpretations include prior arguments that theta and alpha rhythms result from tightly controlled cortico‐thalamic‐cortical signaling loops (Hindriks and van Putten 2013; Lopes da Silva et al. 1973; Nestvogel and McCormick 2022), and recent observations that theta contributing to mediofrontal ERPs could be modeled as a series of inputs to the prefrontal cortex from thalamus (Diesburg et al. 2023). These results are also consistent with recent descriptions of theta and alpha as oscillatory traveling waves that propagate along cortico‐cortical connections (Alamia and VanRullen 2019; Nunez and Srinivasan 2006a; Zhang et al. 2018). These traveling oscillations are thought to have implications for the generation of evoked potentials (Klimesch, Hanslmayr, et al. 2007) and even for human consciousness (Nunez and Srinivasan 2006a). Our findings are thus in line with a literature that describes cortical theta and alpha as primarily oscillations, while remaining agnostic as to the chief mechanism generating these oscillations.

Note that here we analyzed TF power after subtracting the phase‐consistent, or evoked, portion of the signal, and as such we focus on phase‐inconsistent or “induced” activity following the taxonomy established by Galambos (Galambos 1992). This choice was made because there are posited mechanisms of generating ERP components that are neither fully oscillatory nor aperiodic, including simple additivity (Cho et al. 2021; Min et al. 2007; Sauseng et al. 2007), which would be incompatible with our parameterization analysis. As such, our results do not imply anything about the ERP except that it is not the driver of the observed parameterized power differences or correlations. It is possible this approach underestimates certain oscillatory and aperiodic contributions to stimulus processing, since either changes in oscillatory or aperiodic power could give rise to an ERP. This approach also ignores the possibility of evoked activity generated by phase resetting of ongoing EEG oscillations (Brandt 1997; Hanslmayr et al. 2007; Klimesch, Sauseng, et al. 2007; Min et al. 2007; Rawls, Miskovic, and Lamm 2020; Sauseng et al. 2007). We note that in particular, the loss‐gain differences for mediofrontal theta oscillations were dramatically reduced following subtraction of the evoked potential, implying that a large part of the theta‐band neural response to loss feedback is phase‐locked (see Supporting Information). Furthermore, previously observed delta increases for congruent and gain trials (compared with incongruent and loss trials) coinciding with the early portion of the P3 were not observed in our primary analysis, but were observed in data without subtracting the ERP from single trials (Supporting Information), implying that these delta effects are due to influences of the ERP as well.

Many oscillatory phenomena post‐stimulus are only present for two to three cycles of an oscillation. These short‐lived bursts may reflect genuine rhythmic activity, but in other cases they could have other mechanisms. Here, we are confident referring to these events as oscillations because in the plots of conditions‐averaged power, it is clear that mediofrontal theta oscillations are present during the baseline and also post‐stimulus processing (Figure 2). In conducting ERP subtraction, we removed one of these potential non‐oscillatory mechanisms. ERP subtraction in single trials is not a foolproof method, and in some cases can introduce spurious effects. However, we show evidence that all major results replicate in data without subtraction of the evoked potential. Future parameterization methods that can accurately parameterize the multiple biophysical mechanisms giving rise to the ERP are needed to overcome this limitation and determine whether any portion of the ERP might be oscillatory in nature.

### 4.3 Role of Aperiodic Activity During Cognitive Tasks

Parameterization of time‐frequency activity during two common tasks probing cognitive control and reinforcement processing unexpectedly revealed aperiodic differences between congruent and incongruent trials and between gain and loss trials. During the need for cognitive control, central activity in beta frequency ranges was higher for congruent than for incongruent trials, and this difference was primarily ascribed to aperiodic, rather than oscillatory, differences. This implies that aperiodic activity in the range of 15–30 Hz is responsive to cognitive control needs, challenging common assumptions that all or nearly all EEG task time‐frequency responses are oscillatory. A previous study (Gyurkovics et al. 2022) indicated that aperiodic activity changed following stimulus presentation, generally arguing for steeper spectral exponents in trials with higher attentional requirements. This finding was echoed by Zhang et al. (2023), who reported higher aperiodic exponents during the need for inhibitory control. We observed more negative aperiodic beta activity in incongruent trials, corroborating these findings. Intriguingly, aperiodic differences were also observed during reinforcement processing, but these differences were prominent at lower frequencies through the highest measured frequency (30 Hz) and always indicated higher activation for loss than gain trials. However, note that unlike the flanker task, this activation increase was more prominent at lower frequencies, implying that high‐ and low‐frequency aperiodic neural activity can be differentially modulated.

Aperiodic exponent (slope) changes have been argued as reflecting the balance of excitation and inhibition in underlying neuronal populations (Ahmad et al. 2022; Gao et al. 2017; Martínez‐Cañada et al. 2023). The initial study that suggested the aperiodic exponent could be viewed as a marker of E/I balance argued that this relationship emerged in frequencies around 40–70 Hz (Gao et al. 2017). As EEG consists of mostly lower‐frequency power, the aperiodic exponent as measured by EEG might not necessarily reflect E/I balance as conceptualized by Gao et al. (2017). This study also modeled excitation as resulting from only AMPA currents. Excitation in actuality arises mostly from a combination of AMPA and NMDA currents. These two receptor types have different membrane time constants, so AMPA and NMDA‐mediated excitation should impact different points of the aperiodic power spectrum. Based on membrane time constants, we suggest that higher‐frequency aperiodic condition differences (beta and gamma) could be linked to changes in inhibitory (GABA‐ergic) neurotransmission, and lower‐frequency aperiodic condition differences (e.g., delta) could be linked to changes in NMDA‐mediated excitation. Improved modeling work will be required to tease apart these details.

### 4.4 Limitations and Future Directions

Our approach of using short‐time Fourier transform, rather than Morlet wavelets or a similar approach, limits the temporal resolution of our results. Utilizing higher‐resolution methods for time‐frequency decomposition could help gain a more fine‐grained understanding of these dynamics. We were also limited in our low‐frequency accuracy because of the use of STFTs, which might have impacted our abilities to parameterize delta activity. Other current algorithmic issues, including a poor ability to fit common aperiodic “knees” in the delta range, also limit interpretation of delta‐band results. However, STFT has superior noise cancellation properties, and these properties correspond to especially high accuracy in time‐frequency parameterization (Wilson et al. 2022). We also parameterized trial averages, but oscillations necessarily occur in single trials. However, the computational overhead and the significantly reduced accuracy of single‐trial parameterization necessitated this approach. Future time‐frequency analyses capable of providing enhanced single‐trial data quality, or improved power spectral modeling approaches, will likely be needed to resolve these potential concerns. Finally, we tested whether parameterized oscillations correlated with traditional baseline‐corrected power values using percent change from baseline. We acknowledge that slightly different numerical results might be obtained using dB or other baseline correction methods; however, these methods usually yield very similar results when applied to the same data (Cohen 2014). Finally, our results have implications for the common use of single‐trial regression with EEG data. Sometimes it is suggested that in single‐trial regression, the intercept absorbs 1/f activity. However, the intercept of a regression is the average over all observations, so the intercept of a regression can only be said to absorb aperiodic activity if it is the same over all trials. Here, we provide evidence that this is not the case, and as such our results suggest that aperiodic activity could be included in previous single‐trial regression analyses.

### 4.5 Deviations From Registered Report Plans

We took care to follow the plans laid out in the original Stage 1 Registered Report. Nevertheless, changes were made during data analysis and manuscript preparation, which are summarized here. First, as reported in methods, we were unable to analyze two subjects from the gambling paradigm, and we were able to analyze an additional two subjects from the flanker paradigm, slightly adjusting our sample sizes. Second, our Stage 1 registered report did not include subtraction of the evoked portion of the signal. However, a reviewer identified this as a potential limitation, and we added this analysis. Finally, we planned to consider not only the primary condition of the gambling task (Gain‐Loss) but also the secondary dimension (Low‐High Outcome Value). We ran preliminary analyses of condition differences in oscillatory and aperiodic power and observed only sporadic and weak condition differences between point values within Loss and Gain (Supporting Information). The choice was made to analyze only the primary dimension of the gambling task. All other registered plans were closely followed.

### 4.6 Conclusions

Our registered report provides both applied and theoretical contributions. We describe the decomposition of task time‐frequency data into orthogonal oscillatory and aperiodic components, and we also provide easy‐to‐use code for other researchers to do so bundled into the NeuroFreq (Rawls 2023) software. We corroborate previous claims that mediofrontal theta increases during the need for cognitive control and during loss processing, and parietal alpha suppression during the need for control, are essentially oscillatory brain responses. Yet we also showed unexpected, time‐resolved brain differences in aperiodic power, corresponding to broadband increases in power for congruent and loss trials. These time‐dependent shifts in aperiodic power undermine previous claims that baseline correction recovers oscillations free of aperiodic contamination—an assertion supported by our correlation analyses indicating significant remaining correlations between aperiodic power and baseline‐corrected power. We suggest that future directions should include improved biophysical modeling to understand the neurophysiological basis of oscillatory and aperiodic currents in EEG. Ultimately, a richer consideration of the biophysical underpinnings of these patterns of activity will lead to our ability to isolate, and even hopefully intervene on, the neural mechanisms that are causal for cognitive control and learning.

## Author Contributions

E.R.: conceptualization, methodology, software, validation, formal analysis, investigation, writing – original draft, writing – review and editing, visualization, data curation, funding acquisition. S.R.S.: resources, data curation, writing – review and editing, project administration, funding acquisition.

## Conflicts of Interest

The authors declare no conflicts of interest.

## Supporting information


Data S1.

## Data Availability

All code is publicly available. Time‐frequency analysis, evoked potential subtraction, averaging, and baseline correction, spectral parameterization, statistics, and plotting used the publicly available NeuroFreq (Rawls 2023) toolbox https://github.com/erawls‐neuro/NeuroFreq_public. Code for time‐frequency parameterization was adapted from BrainStorm software (Tadel et al. 2011) as described by Wilson et al. (2022). We developed a wrapper for running this parameterization within NeuroFreq, and the underlying functions as implemented in BrainStorm were unchanged. Code for statistical tests used the publicly available ept‐tfce toolbox (Mensen and Khatami 2013). We developed wrappers for using ept‐tfce within NeuroFreq, and the underlying mex‐files are unchanged. If readers apply any methods from this publication, they should cite the appropriate original developers (Mensen and Khatami 2013; Tadel et al. 2011; Wilson et al. 2022), the NeuroFreq toolbox (Rawls 2023), and this manuscript. All data are publicly available. Raw EEG data, summary TF data, TFCE output, and scripts to replicate all statistical tests and plots in the manuscript are available at the Open Science Framework as part of the preregistration associated with this Registered Report: https://osf.io/6mwyu/.
