# Supplementary material for: Oscillatory and Aperiodic Contributions to EEG Event‐Related Time‐Frequency Metrics During Cognitive Control and Reinforcement Processing: A Registered Report
Source: Psychophysiology. 2025 Jun 3;62(6):e70073. doi: 10.1111/psyp.70073 (PMC12134716; doi:10.1111/psyp.70073)
Supplement: Supplementary file 1 — Data S1. [file PSYP-62-e70073-s001.docx]

**SUPPLEMENT to:** Oscillatory and Aperiodic Contributions to EEG Event-Related Time-Frequency Metrics During Cognitive Control and Reinforcement Processing: A Registered Report

**Authors:** Eric Rawls^12^, Scott Sponheim^23^

**Affiliations:** ^1^Department of Psychology, University of North Carolina Wilmington, ^2^Department of Psychiatry and Behavioral Sciences, University of Minnesota, ^3^Minneapolis Veterans Affairs Health Care Services (VAHCS)

**ORCID IDs:** Eric Rawls [
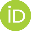
](https://orcid.org/0000-0002-4852-9961) <https://orcid.org/0000-0002-4852-9961>, Scott Sponheim [
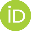
](https://orcid.org/0000-0002-2782-0856) <https://orcid.org/0000-0002-2782-0856>

**Correspondence:** Eric Rawls, [rawls](mailto:rawls017@umn.edu)e@uncw.edu

**1 Supplemental Results**

A primary departure from our registered analysis plan is that we proposed to analyze a classic gambling task using factors of Outcome (gain, loss) and Value (25, 5). Instead we analyzed the task using only a factor of outcome, since we observed nearly no condition differences between high and low value outcomes in the parameterized TF data. Here we present analyses of Value within loss and gain outcomes, to corroborate this. Statistics from this analysis are reported in *Figure S1*.

Second, given the potential for the event-related potential to be generated by mechanisms other than solely oscillations or brain aperiodic activity, and which is not easily parameterizable, our primary analyses considered time-frequency power after the phase-consistent or “evoked” portion of the power was subtracted leaving only induced power. Here we present complementary results without subtracting the evoked part of the signal, in part for completeness and in part to show that results do not at any point categorically depend on whether or not the evoked potential is present in the signal. That is, all of the results shown here indicate the same patterns as those shown in the main manuscript. Statistics from these analyses are presented in the same order as in the main manuscript, and are reported in *Figures S2-S8.*

*
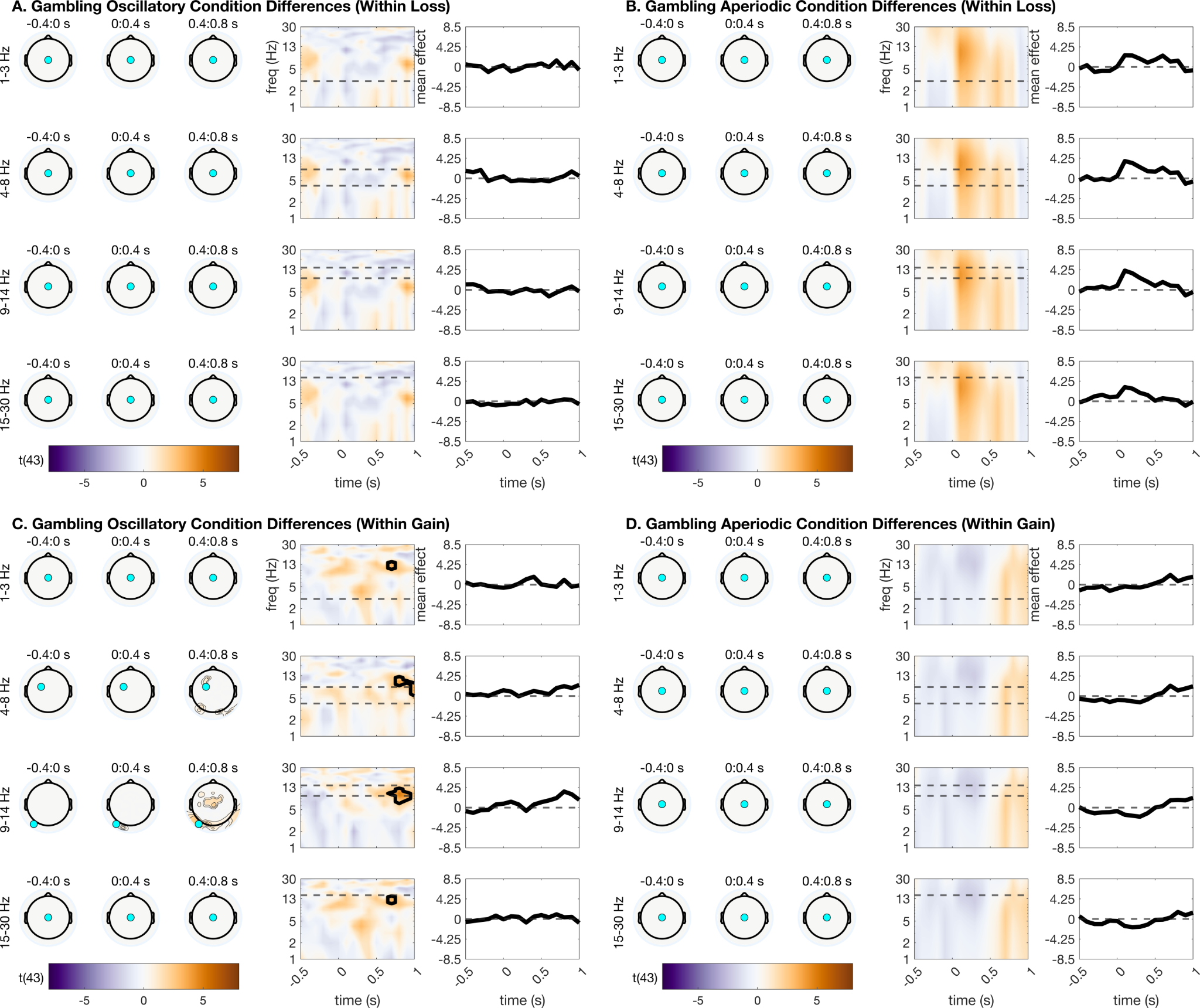
*

*Figure S1. Gambling task condition differences by Value, separately within Gain and Loss outcomes. Only very minor and sporadic results were observed, prompting our collapsing over these conditions in the main manuscript. A: No condition differences for oscillatory power within loss trials. B: No condition differences in aperiodic power within loss trials. C: Minor alpha-band oscillatory enhancements almost 800 ms following high-valued outcomes (compared to low). D: No condition differences for aperiodic power within gain trials.*

*
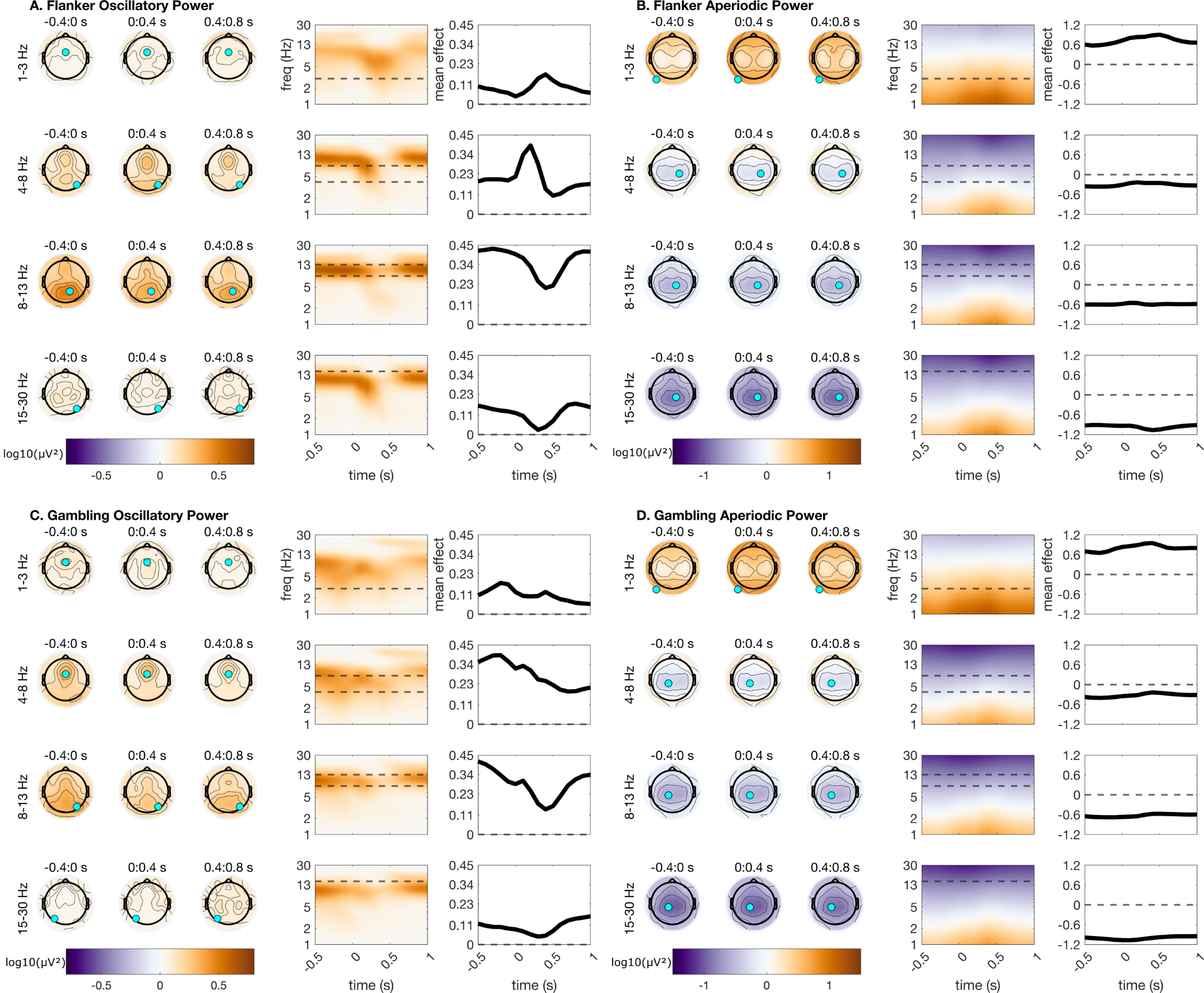
*

*Figure S2. Condition-averaged power from the Flanker and gambling paradigms. All topographic plots are divided by frequency bin, where delta = 1-3 Hz, theta = 4-8 Hz, alpha = 9-14 Hz, and beta = 15-30 Hz. Note that statistical tests clustered over frequency neighbors rather than using a priori bins, so this is merely for plotting purposes. A: Oscillatory power for flanker. B: Aperiodic power for flanker. C: Oscillatory power for gambling. D: Aperiodic power for gambling.*

*
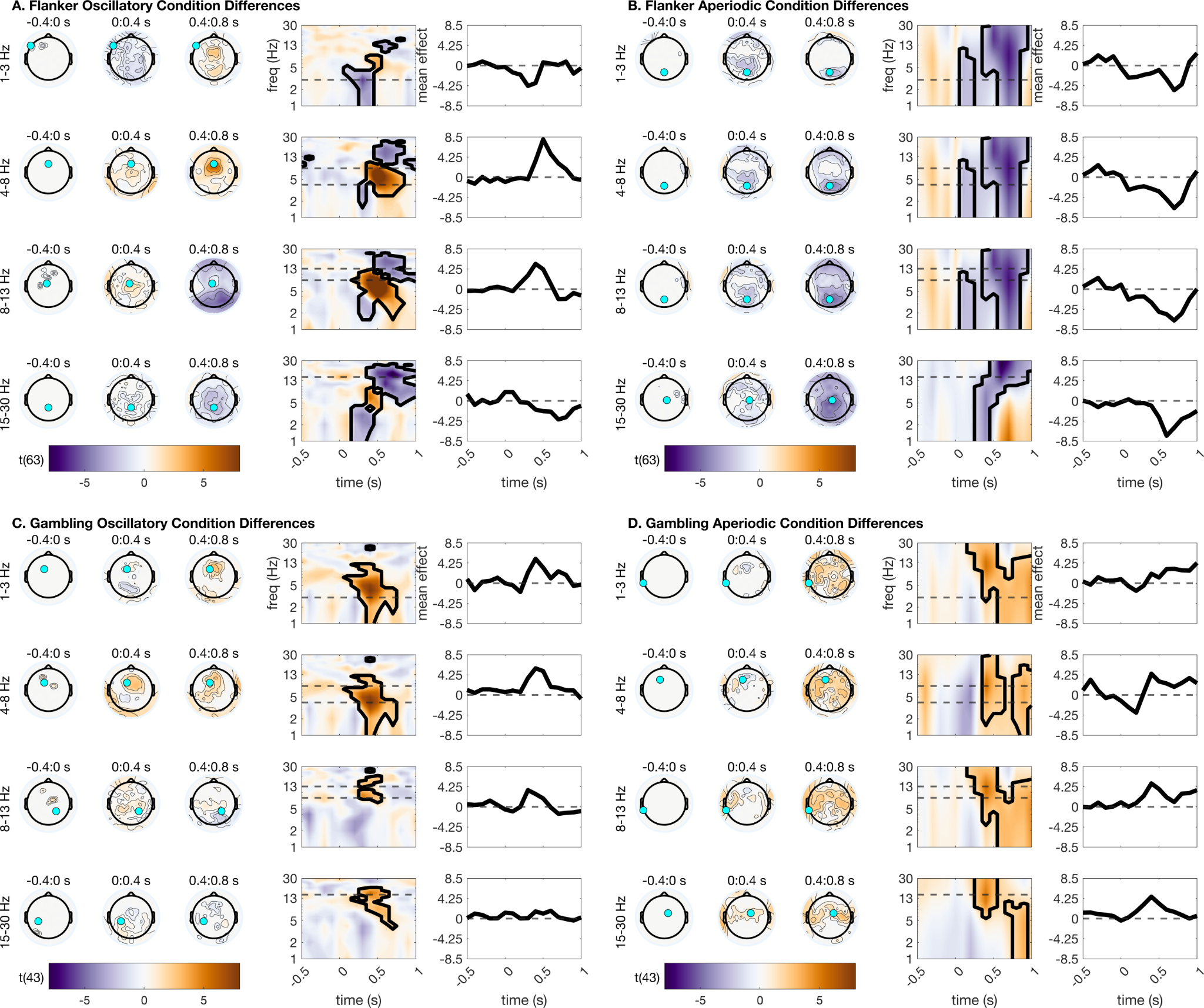
*

*Figure S3. Condition-difference power from the flanker and gambling paradigms. Flanker plots show incongruent-congruent, and gambling plots show loss-gain. Topographic plots are masked by significance, so any contours outline significant effects. Likewise, contours on TF surfaces highlight significant clusters. A: Oscillatory condition differences for flanker. B: Aperiodic condition differences for flanker. C: Oscillatory condition differences for gambling. D: Aperiodic condition differences for gambling.*

*
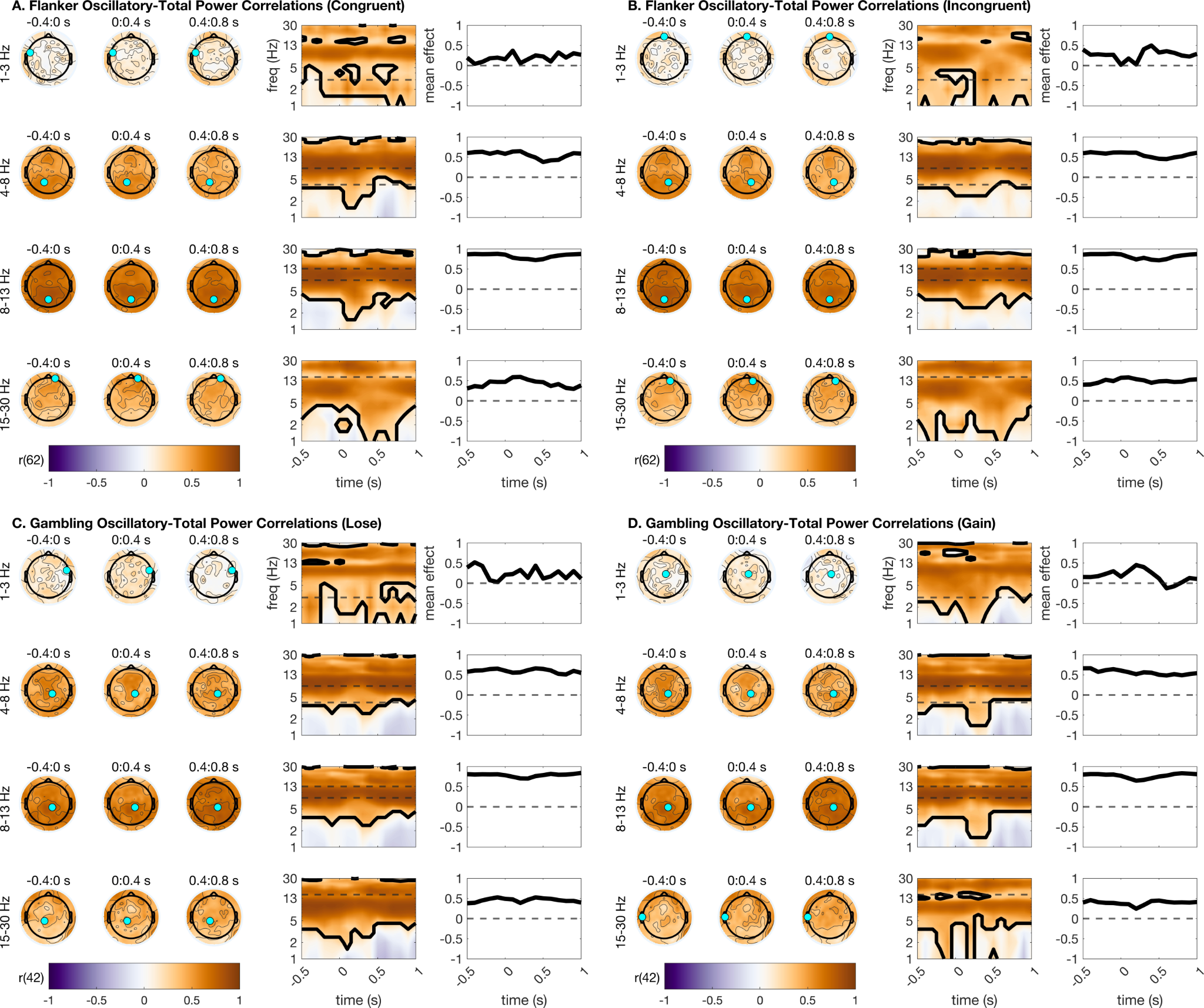
*

*Figure S4. Total power correlations with parameterized oscillation estimates. Correlations were high across alpha, and to a lesser extent theta and beta, bands. Topographic plots are masked by significance, so any contours outline significant effects. Likewise, contours on TF surfaces highlight significant clusters. A: Total power-oscillatory correlations for the flanker task, congruent trials. B: Total power-oscillatory correlations for the flanker task, incongruent trials. C: Total power-oscillatory correlations for the gambling task, loss trials. D: Total power-oscillatory correlations for the gambling task, gain trials.*

*
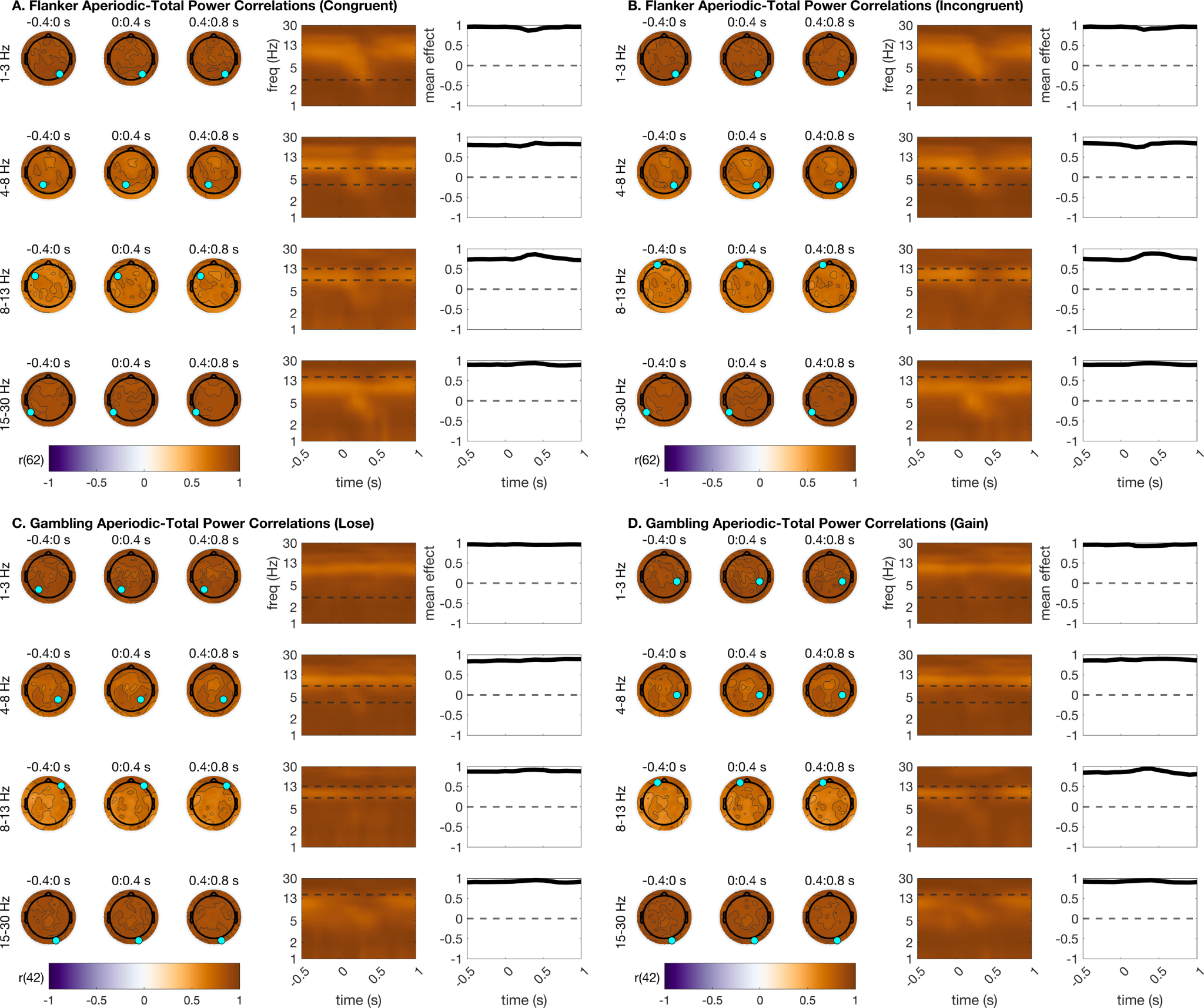
*

*Figure S5. Total power correlations with parameterized aperiodic estimates. Correlations were uniformly extremely high. A: Total power-aperiodic correlations for the flanker task, congruent trials. Note that all points are highly significant so no masking is needed. B: Total power-aperiodic correlations for the flanker task, incongruent trials. C: Total power-aperiodic correlations for the gambling task, loss trials. D: Total power-aperiodic correlations for the gambling task, gain trials.*


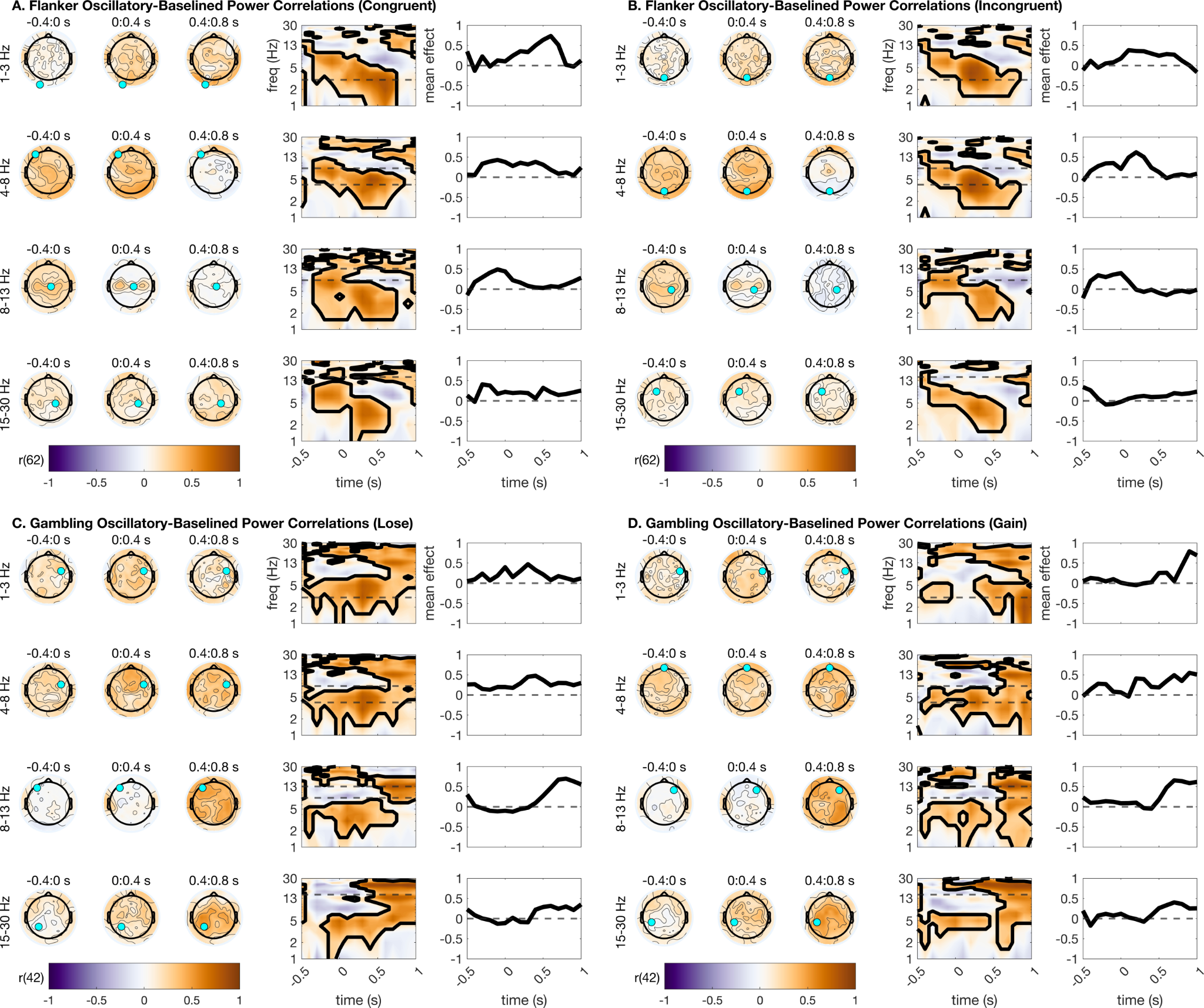


*Figure S6. Baselined power correlations with parameterized oscillation estimates. Correlations were high across theta, alpha, and beta ranges. Topographic plots are masked by significance, so any contours outline significant effects. Likewise, contours on TF surfaces highlight significant clusters. A: Baselined power-oscillatory correlations for the flanker task, congruent trials. B: Baselined power-oscillatory correlations for the flanker task, incongruent trials. C: Baselined power-oscillatory correlations for the gambling task, loss trials. D: Baselined power-oscillatory correlations for the gambling task, gain trials.*


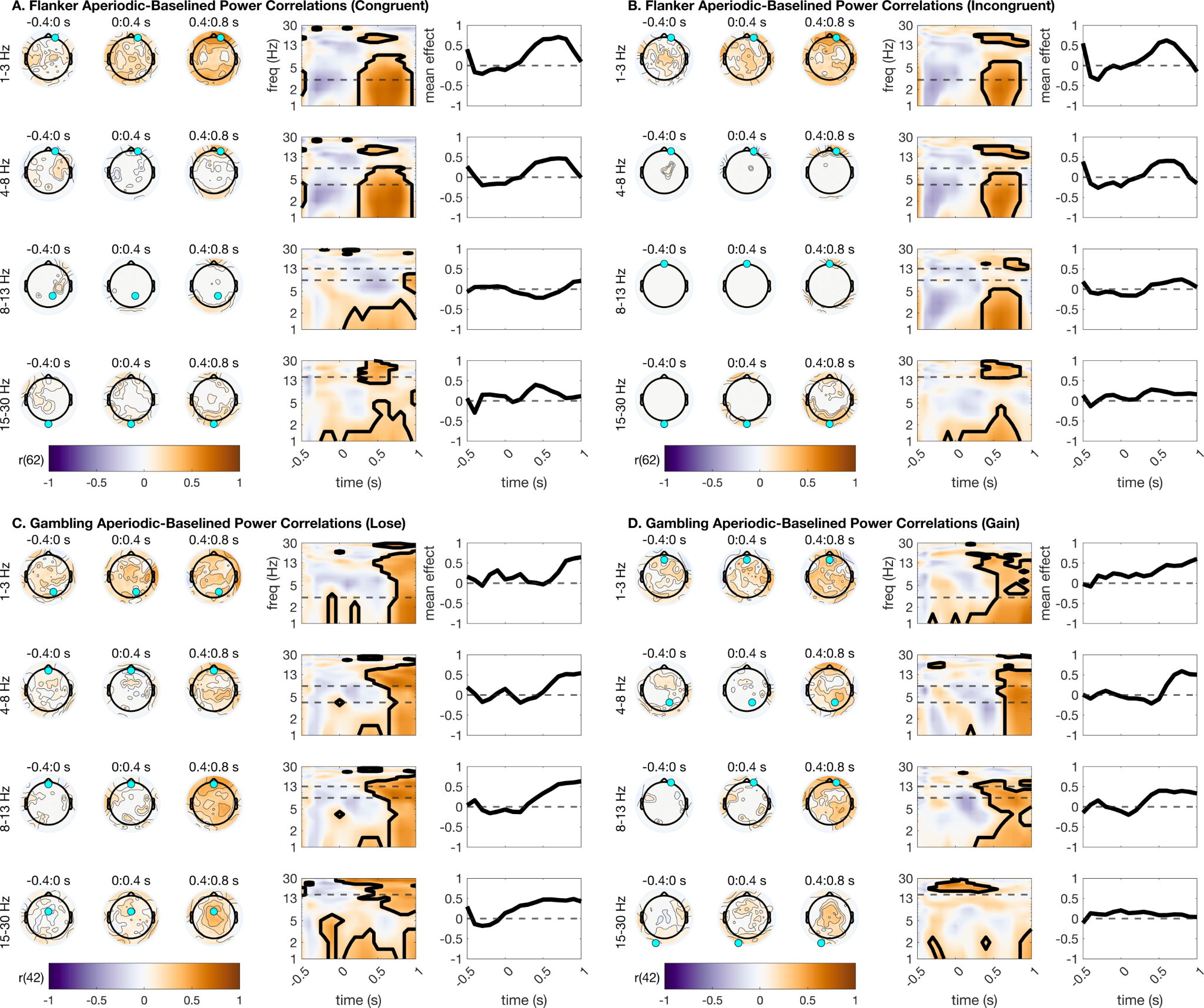


*Figure S7. Baselined power correlations with parameterized aperiodic estimates. Correlations were high across delta, alpha, and beta ranges. Topographic plots are masked by significance, so any contours outline significant effects. Likewise, contours on TF surfaces highlight significant clusters. A: Baselined power-aperiodic correlations for the flanker task, congruent trials. B: Baselined power-aperiodic correlations for the flanker task, incongruent trials. C: Baselined power-aperiodic correlations for the gambling task, loss trials. D: Baselined power-aperiodic correlations for the gambling task, gain trials.*

*
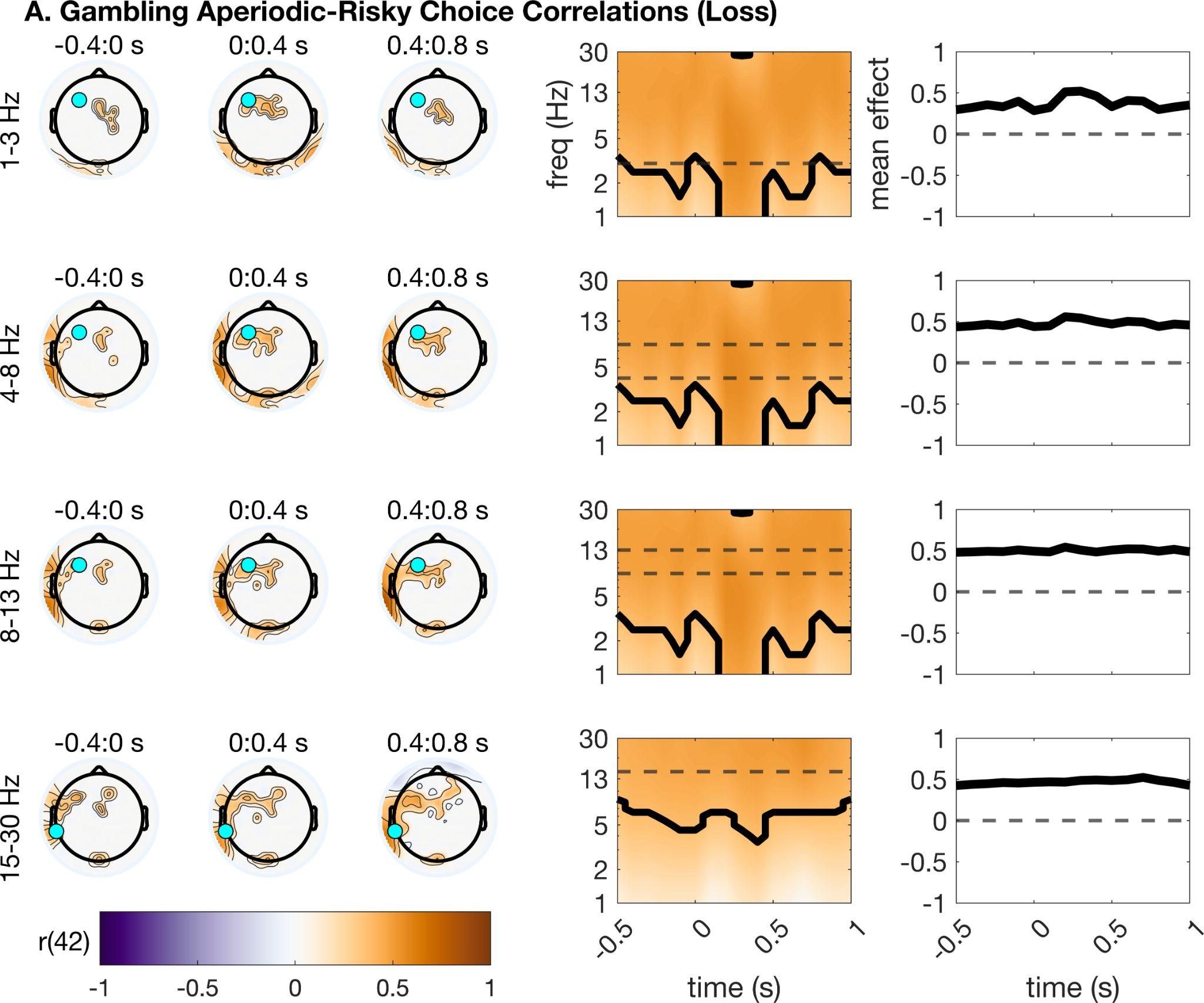
*

*Figure S8. Correlations between parameterized gambling EEG and behavior. Topographic plots are masked by significance, so any contours outline significant effects. Likewise, contours on TF surfaces highlight significant clusters. A: Oscillatory-risk correlation (loss). B: Aperiodic-risk correlation (loss). C: Oscillatory-risk correlation (gain). D: Aperiodic-risk correlation (gain).*


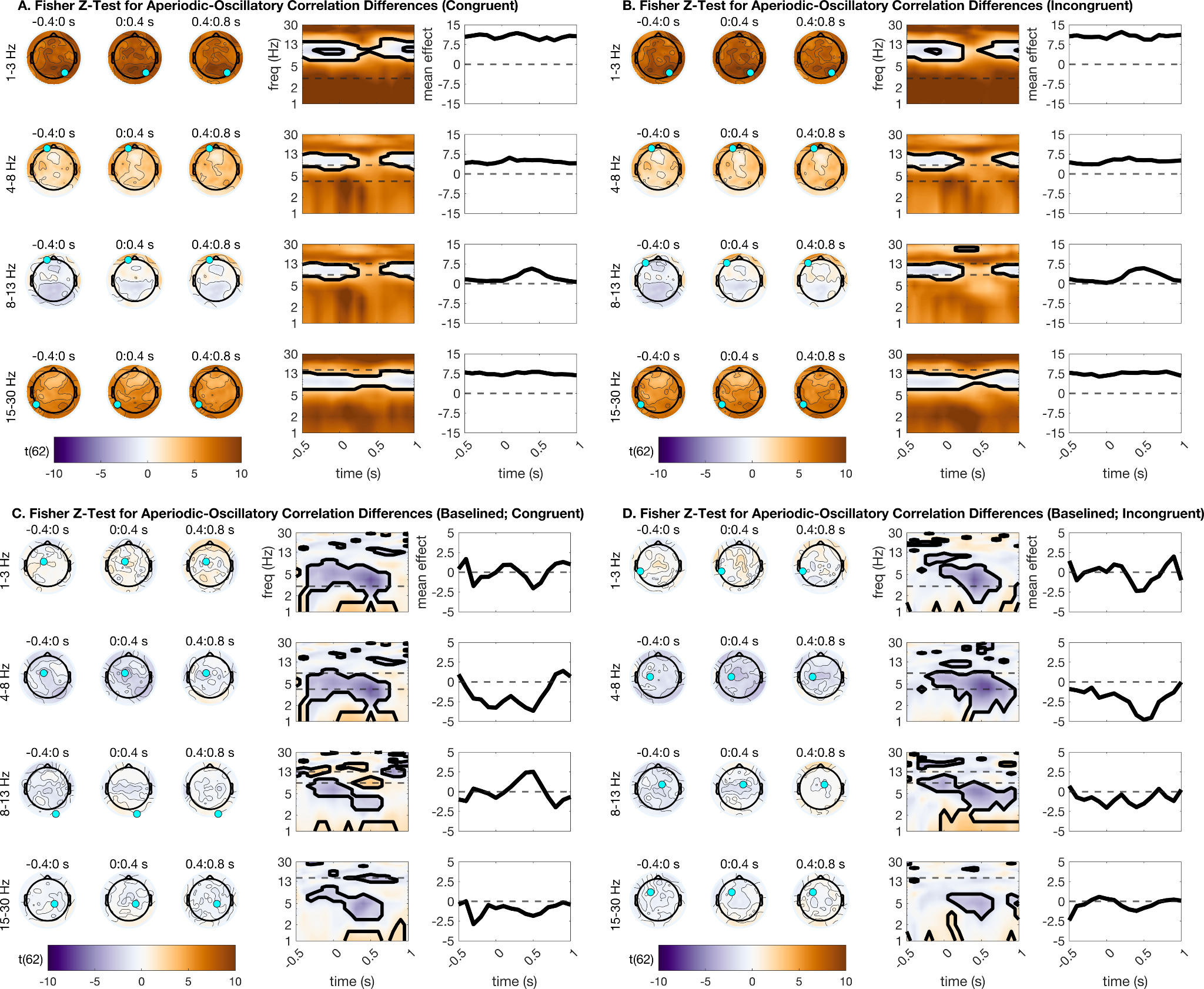


*Figure S9. Flanker Task. Results of a series of Fisher z-tests, comparing the correlations of total power (A & B) and baseline-corrected power (C & D) with parameterized oscillations and aperiodic power. Color maps indicate the subtraction of oscillatory correlations from aperiodic correlations, and as such warm colors indicate areas of the time-frequency space where total power correlates more highly with aperiodic than with oscillatory estimates. Cool colors thus signify regions where power is correlated more highly with oscillatory than aperiodic estimates. A & B: Prior to baseline correction, aperiodic power correlated very highly with total power, except for in alpha ranges. C & D: After baseline correction, oscillatory power correlated more highly with baseline-corrected power than aperiodic power did. This effect was particularly strong in theta frequency ranges, implying that baseline correction yields theta estimates that are highly correlated with parameterized oscillations.*

*
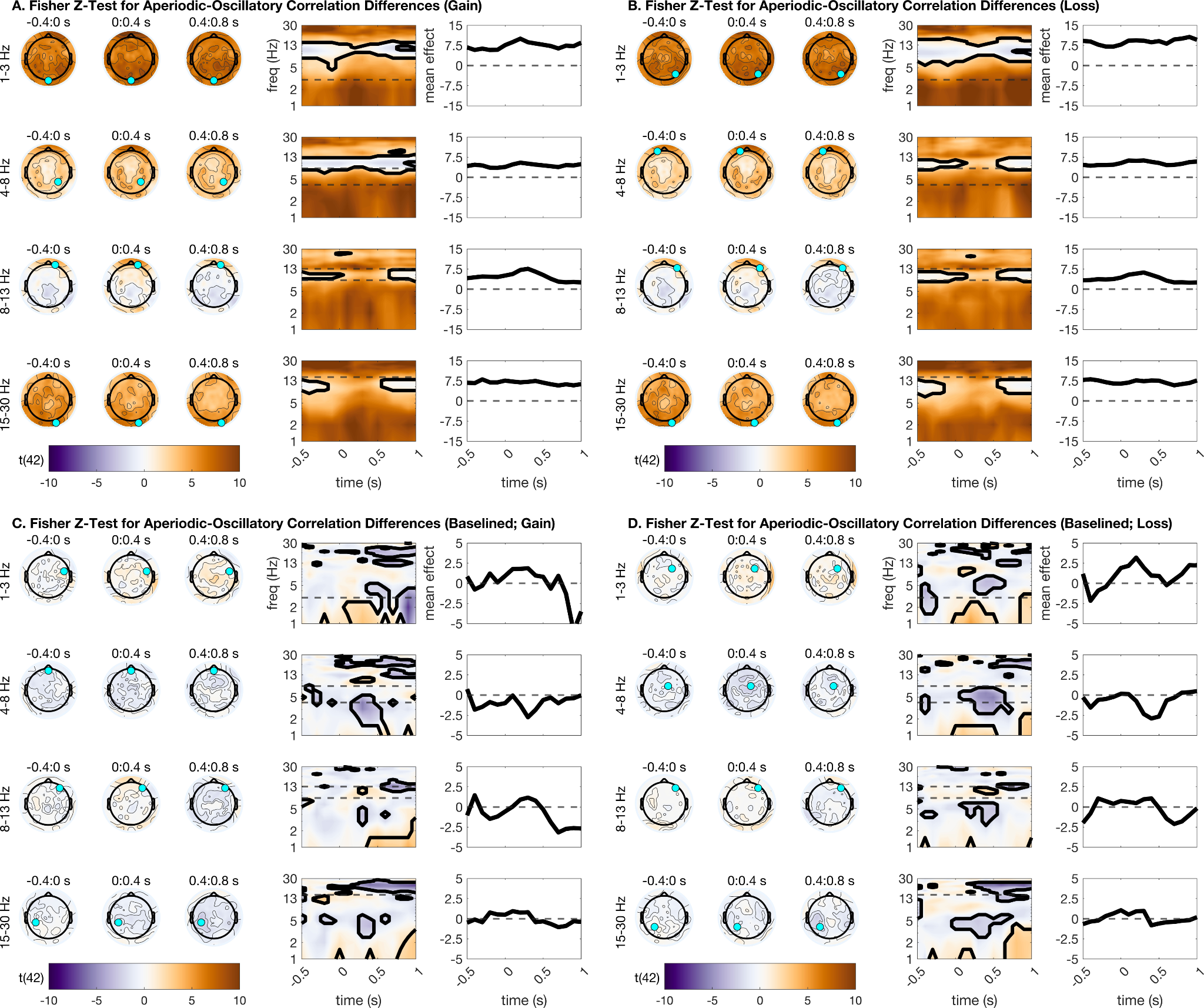
*

*Figure S10. Gambling Task. Results of a series of Fisher z-tests, comparing the correlations of total power (A & B) and baseline-corrected power (C & D) with parameterized oscillations and aperiodic power. Color maps indicate the subtraction of oscillatory correlations from aperiodic correlations, and as such warm colors indicate areas of the time-frequency space where total power correlates more highly with aperiodic than with oscillatory estimates. Cool colors thus signify regions where power is correlated more highly with oscillatory than aperiodic estimates. A & B: Prior to baseline correction, aperiodic power correlated very highly with total power, except for in alpha ranges. C & D: After baseline correction, oscillatory power correlated more highly with baseline-corrected power than aperiodic power did. This effect was particularly strong in theta frequency ranges, implying that baseline correction yields theta estimates that are highly correlated with parameterized oscillations.*
